# Peptidase specificity from the substrate cleavage collection in the *MEROPS* database and a tool to measure cleavage site conservation

**DOI:** 10.1016/j.biochi.2015.10.003

**Published:** 2016-03

**Authors:** Neil D. Rawlings

**Affiliations:** Wellcome Trust Sanger Institute and the EMBL-European Bioinformatics Institute, Wellcome Trust Genome Campus, Hinxton, Cambridgeshire, CB10 1SA, UK

**Keywords:** Peptidase, Substrate, Specificity, Cleavage, Binding pocket, Scissile bond

## Abstract

One peptidase can usually be distinguished from another biochemically by its action on proteins, peptides and synthetic substrates. Since 1996, the *MEROPS* database (http://merops.sanger.ac.uk) has accumulated a collection of cleavages in substrates that now amounts to 66,615 cleavages. The total number of peptidases for which at least one cleavage is known is 1700 out of a total of 2457 different peptidases. This paper describes how the cleavages are obtained from the scientific literature, how they are annotated and how cleavages in peptides and proteins are cross-referenced to entries in the UniProt protein sequence database. The specificity profiles of 556 peptidases are shown for which ten or more substrate cleavages are known. However, it has been proposed that at least 40 cleavages in disparate proteins are required for specificity analysis to be meaningful, and only 163 peptidases (6.6%) fulfil this criterion. Also described are the various displays shown on the website to aid with the understanding of peptidase specificity, which are derived from the substrate cleavage collection. These displays include a logo, distribution matrix, and tables to summarize which amino acids or groups of amino acids are acceptable (or not acceptable) in each substrate binding pocket. For each protein substrate, there is a display to show how it is processed and degraded. Also described are tools on the website to help with the assessment of the physiological relevance of cleavages in a substrate. These tools rely on the hypothesis that a cleavage site that is conserved in orthologues is likely to be physiologically relevant, and alignments of substrate protein sequences are made utilizing the UniRef50 database, in which in each entry sequences are 50% or more identical. Conservation in this case means substitutions are permitted only if the amino acid is known to occupy the same substrate binding pocket from at least one other substrate cleaved by the same peptidase.

## 1 Introduction

In 2007, Barrett & Rawlings [1] proposed a list of criteria to distinguish one peptidase from another. To be considered different, any one of the following bioinformatics tests can be applied: the two peptidases have similar biochemical characteristics but unrelated sequences; the two peptidases have related sequences but different biochemical properties, different domain architectures or the domains are in a different order; or the two peptidases have greater than 50% sequence identity but are derived from nodes on a phylogenetic tree that are not adjacent. In addition, the following biochemical tests can be applied to distinguish two peptidases: the peptidases act under significantly different conditions; the peptidases have different post-translational modifications; the peptidases are sensitive to different inhibitors; the peptidases act on different substrates, or if they act on the same substrates then the cleavage positions are different. It is the last two criteria with which this paper is concerned.

A peptidase cleaves a substrate at the scissile bond, and substrate residues either side of this bond are known as P1 and P1′. Residues towards the N-terminus of the substrate are on the non-prime side, and are numbered P1, P2, P3, P4 and so on. Residues towards the C-terminus are on the prime side and are numbered P1′, P2′, P3′, P4′ and so on. A substrate binding pocket in the peptidase that accommodates a substrate residue is named according to the position the residue occupies in the substrate, except that the “P” is replaced by an “S”. So the S1 binding pocket accommodates the P1 residue, and the S4′ binding pocket accommodates the P4′ residue [2].

A collection of substrate cleavages has been assembled from the scientific literature, annotated, cross-referenced where applicable to the UniProt protein sequence database, and included within the MEROPS database. This collection was originally derived from the CD-ROM version of the first edition of the *Handbook of Proteolytic Enzymes* (1998) [3], which also included a search facility to find the peptidases able to cleave a substrate at a particular position. By knowing where in proteins, peptides or synthetic substrates cleavages occur, it is possible to postulate the specificity of a peptidase. By knowing which amino acids can occupy each substrate binding position, it is also possible to infer whether or not cleavage of a substrate at a particular position is likely to be physiologically relevant from an alignment of protein sequences of closely-related orthologues.

The MEROPS substrate cleavage collection has been widely used to predict cleavages in substrates (for a review see Song et al. (2011) [4]), and to predict what peptidase may be responsible for a known cleavage, for example PROSPER [5]. The MEROPS collection has also been used for the mapping of the human degradome and prediction of “cleavage entropy” as an overall measure of peptidase specificity [6], as well as in the development of the “protease web”, the network of peptidase, substrate and inhibitor interactions [7].

This paper describes the MEROPS substrate cleavage collection and the various displays present on the MEROPS website (http://merops.sanger.ac.uk) which aid in understanding peptidase specificity and the processing and degradation of a protein substrate. In order to help determine whether or not a cleavage is physiologically relevant, a service is also described where a user can upload substrate cleavages and receive by E-mail an analysis to show how well conserved, in terms of peptidase binding, each cleavage is.

## 2 Materials and methods

### 2.1 Identification of peptidases, homology searching, sequence alignment and phylogenetic tree generation

A peptidase species was defined according to the principles established in Barrett & Rawlings (2007) [1]. The methods for homology searching, family building, and generation of protein sequence alignments and phylogenetic trees are the same as those described in Rawlings et al. (2014) [8]. In brief, the following methods were used. Only the peptidase domain was used for sequence searching and sequence alignment. For each family a *type example* was chosen and for each peptidase species a *holotype* was chosen. The type example and holotype were usually the sequence of the best characterized peptidase in the family or protein species, respectively. A BlastP search [9] of the NCBI non-redundant protein sequence database was performed, using the family type example sequence. Sequences retrieved with an E value of 0.01 or less were considered homologues and included in the family. To find more distant homologues, a HMMER search [10] was performed using a ClustalW alignment [11] of a selection of sequences from the family that included an example from every phylum for which there was a representative. Sequence alignments were built using MAFFT [12]. Phylogenetic trees were built from the family sequence alignment using QuickTree [13].

### 2.2 Manual substrate cleavage curation

The scientific literature was searched manually for substrate cleavage sites by peptidases. Data were acquired from over 7280 references. The following data were collected, transformed as required and stored in a MySQL database. From the name of the peptidase as given by the authors of the publication, a *MEROPS* identifier and, if possible, a MERNUM indicating the source organism, were assigned. From the name of the substrate and its source, a UniProt accession was assigned where possible, and the name recommended by UniProt was stored in the MySQL database, unless the substrate was a peptide or was processed, in which case a peptide name or a name to indicate that processing had occurred was stored (for example, “Met-enkephalin” would be stored in preference to “pro-opiomelanocortin” if the substrate was just the peptide). Where more than one UniProt entry existed, the annotated SwissProt accession, name and sequence where used in preference. Where isoforms derived from alternative initiation and alternative splicing were indicated in the UniProt database entry, the sequence chosen as the representative sequence by UniProt was selected unless the original publication indicated that a particular isoform had been used. There was no attempt to map a cleavage to all isoforms on the presumption that a change in sequence could lead to a change in cleavage position. The cleavage position (the position of the P1 residue in the substrate) was converted to the equivalent residue number from the respective UniProt entry. Up to four residues either side of the scissile bond (residues P4 to P4′) were stored for each cleavage. The residue range of the substrate used compared to the sequence in the UniProt entry was also stored. This allowed for annotation of peptide substrates derived from full-length proteins and processing events, such as removal of signal and transit peptides and precursor sequences. The CDC checksum for the UniProt entry was also stored so that any changes to the sequence could be identified subsequently. Kinetic data (*K*_m_, *K*_cat_, and/or *K*_m_/*K*_cat_) were stored where available. Annotations to indicate how the peptidase and cleavage position were identified were also stored. The initials of the curator and the date the cleavage was collected were also stored. The reference was stored in a Reference Manager database (Thomson Reuters) and the PubMed accession was obtained and stored where possible. Any additional data that affected where cleavage occurred, such as reactions conditions, where stored as a comment in the MySQL database.

To ensure that curation was consistent, a Perl program was written to aid cleavage data collection and storage. The user (either the author or a summer student) was asked to enter his or her initials; the UniProt accession of the protein substrate in question; the cleavage position; the residue range of the substrate sequence compared to the UniProt entry; the codes for how the cleavage was identified and how the peptidase was identified; whether the cleavage was physiological, non-physiological, pathological or theoretical; whether the substrate was denatured; the reference and its PubMed identifier; and any comment.

Collection of cleavage data from the literature was also outsourced to Molecular Connections, Bangalore, India. Data were returned to the author as an Excel spreadsheet and a pipeline developed to extract data from the spreadsheet and import it into the MySQL database. Existing substrate cleavage collections were also imported into the MEROPS collection. These included data from the CutDB database [14] and the CASBAH database of caspase substrates [15].

A Perl program to check that the P4–P4′ residues around the cleavage position matched the sequence in the UniProt entry was written as a quality control measure and to identify any subsequent changes in the UniProt sequence.

Cleavage data were also stored for the cleavage of synthetic substrates. These were manually entered into the database. For a synthetic substrate it was not possible to map the sequence to a UniProt database entry. The P4–P4′ positions around the scissile bond were stored where possible (many synthetic substrates do not have residues beyond P1′ or P3), including a unique identifier for each N- or C-terminal blocking or reporter group occurring within that range.

In certain cases, it was not possible to map a cleavage to a single enzyme. This most frequently occurred when cleavage was performed by an enzyme complex, such as the proteasome or eukaryotic signal peptidase, or where cleavage might have been performed by one or more enzymes, such as removal of the initiating methionine by either of the eukaryotic methionyl aminopeptidases, or where specificity overlapped, for example caspases-3 and -7. Instead of mapping the cleavage to an individual enzyme, the cleavage was mapped to the peptidase family.

### 2.3 Automated collection of substrate cleavages from SwissProt entries

Entries in the SwissProt section of UniProt are annotated for removal of initiating methionine, signal and transit peptides, and processing events to activate proteins or extract peptides and proteins from polyproteins. These, if not already present in the substrate cleavage collection, were automatically collected using a Perl script. Removal of an initiating methionine was mapped to a MEROPS identifier either for methionyl aminopeptidase (for a prokaryote) or the M24 family (for a eukaryote). Removal of a signal peptidase was mapped to a MEROPS identifier either for signal peptidase or a signal peptidase complex (depending on whether the source species was a prokaryote or a eukaryote). Where the peptidase responsible for the cleavage was not known, the cleavage was not mapped to a specific peptidase or peptidase family. Theoretical cleavages, for example the release of a predicted signal peptide, were not collected.

### 2.4 Automated collection of substrate cleavages from proteomics experiments

High-throughput identification of proteins in a sample is known as “proteomics”. In many proteomics experiments, proteins are digested with a peptidase so that each protein can be identified from the mass and charge (and sometimes sequence) of its peptides. This requires a prior *in silico* digestion of all proteins in the proteome, and the choice of the peptidase used for the digestion is important: the peptidase must have a very simple specificity that is easily predictable. For this reason, trypsin. which cleaves all lysyl and arginyl bonds in a denatured protein, with the exception of except Lys-Pro and Arg-Pro bonds, is frequently used. There are many tools to perform theoretical cleavages, for example PeptideMass (http://web.expasy.org/peptide_mass/, [16]) which predicts cleaves by peptidases such as trypsin, chymotrypsin, peptidyl-Lys metallopeptidase and glutamyl peptidase I as well as cleavages by chemicals such as CnBr. The neXtProt database includes trypsin digestions of all human proteins and indicates peptides unique to a protein and whether a particular peptide is found in many human proteins [17]. The PRIDE database [[18]; http://www.ebi.ac.uk/pride/archive/] stores the results of proteomics experiments where peptides are identified from a sample by mass spectroscopy. There are a number of different techniques employed of which PICS, TAILS, COFRADIC, Subtiligase, ChaFRADIC and N-terminomics are the most well-known; these have been reviewed by Lai et al. [19] and Schlage & auf dem Keller [20]. TAILS [21] and COFRADIC [22] are techniques that have been employed to identify physiological substrates, including naturally occurring N-termini. Frequently, the peptides derived from a protein can provide evidence of post-translational processing, such as removal of an initiating methionine, signal peptide or transit peptide. A Perl program was written to collect peptides and their corresponding UniProt entries from the PRIDE database and check each to see if the N-terminus matched the site of removal of a hypothetical signal or transit peptide. Because aminopeptidases may further trim an N-terminus once a transit peptidase has been removed, for example aminopeptidase P3 removes an unstable amino terminal tyrosine from mitochondrial proteins following removal of the first transit peptide by mitochondrial processing peptidase but before removal of a second transit peptide by mitochondrial intermediate peptidase [23], the cleavage was only added to the MEROPS collection if the peptide identified and the predicted processing event corresponded. Cleavages from digests performed with more unusual peptidases, such as glutamyl endopeptidase for example, were also collected to boost the number of substrates for these peptidases.

In the last decade or so, there have been a number of attempts to determine peptidase specificity or discover physiological substrates by use of proteomics. In a typical proteomics experiment, a sample, often a human cell lysate, is divided into two portions. The first portion is digested only by trypsin (or a similar peptidase with a known and predictable specificity), and the second with trypsin and a peptidase of choice. The peptides generated are identified by mass spectroscopy. The cleavages that occur only in the second portion are the result of the peptidase of choice. If the second portion is digested first by trypsin and then by the peptidase of choice, then a suite of peptides is prepared to investigate the specificity of the peptidase of choice and the cleavages by this peptidase are not physiologically relevant. Alternatively, if the second portion is digested first with the peptidase of choice and then by trypsin, the digested proteins are likely to include physiological substrates. However, some proteins may be digested because the usual physiological barriers to digestion (compartmentalization, differences in pH, *etc*) have been removed in the experiment, and some proteins may be “bystanders” which are cleaved under physiological conditions but the cleavage is non-functional [24]. The results from analyses by software such as Mascot [25] or PeptideProphet [26] are exported to Excel spreadsheets. These are often deposited as material supplementary to the paper, but spreadsheets have also been kindly supplied by the authors. In a given sample, there can be hundreds or even thousands of peptide products, so manual curation is not possible. A Perl program was written to extract the accessions or identifiers, translate them into UniProt accessions, and then determine the residue number for the P1 residue in each cleavage. All of the cleavages from the seminal paper by Schilling & Overall (2008) [27] were collected, including cleavages by trypsin, but for subsequent papers, trypsin cleavages were not collected.

### 2.5 Reliability scores

A “reliability score” is calculated which is an average percentage difference for all substrates of a peptidase over the range P4–P4′. All possible pairwise comparisons are made and the number of differences summed for all comparisons. For substrates with non-standard amino acids and blocking and reporter groups such as those found in synthetic substrates, the unusual residues are replaced with “X” before the comparisons are performed. The sum of differences is divided by the number of comparisons times the number of positions considered. For most endopeptidases, the number of positions considered is eight, because at least of one of each of the positions P4 to P4′ will be filled. However, for aminopeptidases, where P4 to P2 are empty, and carboxypeptidases, where P2′ to P4′ are empty, only five positions are considered. For dipeptidyl-peptidases and peptidyl-dipeptidases only six positions are considered, and for dipeptidases only two. The number of positions considered is calculated and not assumed. A peptidase with a reliability score of greater than 75 means that its substrates are varied in sequence and the calculated preferences are likely to be correct. A peptidase with a score in the range 50–74% had a proportion of substrates with similar sequences and thus the calculated preferences were less reliable, and the calculated preferences for a peptidase with a score of less than 50% should be treated with caution.

## 3 Results and discussion

### 3.1 Number of peptidases

By using the criteria of Barrett & Rawlings (2007) [1], it was possible to distinguish 2457 different peptidase species. A holotype was established for each of these. In addition, there are a further 250 different peptidase activities in the literature for which either no or too little sequence information exists to be able to map any of them to a UniProt accession. The breakdown into different catalytic types is shown in Table 1. More serine peptidases (554 or 20%) were found than for any other catalytic type.

The holotypes and their sequence homologues, 502,782 sequences in total, were found to be distributed amongst 254 families. This total includes 3669 sequences that are classified as asparagine lyases rather than peptidases, because proteolysis is dependent upon cyclization of an asparagine residue to a succinimide which does not involve hydrolysis [28].

### 3.2 Numbers of substrates

The total number of cleavages found for all peptidases was 66,615. This number includes substrates cleaved in the same position by different peptidases. Most of these cleavages (59,276 or 89%) were in peptides or proteins that could be mapped to UniProt identifiers: 5821 (or 9%) were in synthetic substrates. The majority of these cleavages (36,229 or 54%) are non-physiological, but 20,264 (30%) are thought to be physiologically relevant, and a further 1349 (2%) are pathological. The totals include self-cleaving reactions, which in the case of asparagine lyases is all the cleavages identified for this catalytic type.

The breakdown of substrate cleavages per catalytic type is shown in Table 2. Most cleavages (30,711 or 46%) are for serine peptidases, a number boosted by the large number of non-physiological cleavages (21,301 or 32%).

**Fig. 1:**
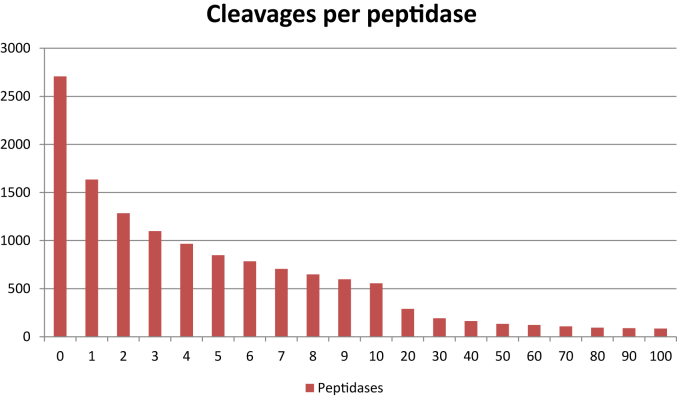
**Cleavages per peptidase**. The bar chart shows the number of known substrate cleavages per peptidase on the Y axis and the count of peptidases with this number of cleavages on the X axis.

Table 3 shows the number of peptidases in each catalytic type for which at least one substrate cleavage is known. The total number is 1700 or 63% of the total number of different peptidases. Comparing the numbers with those in Table 1 shows that for each catalytic type, a substrate cleavage is known for most peptidases. Substrates may be known for any of the other 1007 peptidases, but cleavages were either not found or were not accessible in the literature, or the site of cleavage is unknown. On average a peptidase has 39 substrate cleavages in the MEROPS collection. Table 4 shows the peptidases with 10 or more known cleavages: the large number of non-physiological cleavages for the serine peptidase trypsin, 12,303 of which are derived from the proteomics paper of Schilling & Overall (2008) [27], where it is used for preparation of samples prior to mass spectroscopy analysis, explains the preponderance of cleavages for serine peptidases. To be able to study peptidase specificity and make predictions about where in a protein cleavage might occur, at least 40 cleavages in substrates are required (Robert Pike, personal communication). The number of peptidases with 40 or more cleavages is 163 (or 6% of the total number of peptidases). It is immediately apparent that except for a small number of peptidases, insufficient numbers of cleavages in substrates are known to be able to draw firm conclusions about the specificity of most peptidases. The distribution of cleavages per peptidase is shown in Fig. 1.

There are 36 peptidases with 100 or more known cleavages in physiological substrates. The peptidases with most physiological cleavages are general processing peptidases, such as the animal signal peptidase complex (a complex containing two peptidases with 1879 cleavages) and methionyl aminopeptidase 1 (964 cleavages). Almost all of these cleavages are derived from annotations in the SwissProt section of the UniProt database, and include theoretical cleavages confirmed by proteomics experiments submitted to the PRIDE database. Other peptidases with many physiological cleavages include peptidases involved in protein turnover such as cathepsins E (1553 cleavages) and D (871) and granzyme M (891) almost all of which are derived from proteomics studies [29], [30]. There are 27 peptidases with 200 or more known cleavages in substrates thought not to be physiological. As mentioned above, the use of trypsin to produce peptides for mass-spectroscopy in proteomics experiments means that more cleavages (14,201) are known for this peptidase rather than any other. Large numbers of non-physiological cleavages are also known for peptidases regularly used in manual protein sequencing such as pepsin A (350 cleavages), peptidyl-Lys metallopeptidase (2105), chymotrypsin A (1012), glutamyl peptidase 1 (1273) and lysyl endopeptidase (802). Other peptidases with many non-physiological substrates are again the subjects of proteomic studies: matrix metallopeptidase-2 (2726 cleavages) [27]; human granzyme B (1136) [30], [31]; glutamyl peptidase I (1003) [27]; cathepsins L (965), S (694) and B (486) [32]; meprin beta (891) and alpha subunits [33]; and the RC1339 protein from *Rickettsia conorii* (799) [34]. There are 57 peptidases with 20 or more cleavages in synthetic substrates and neurolysin (139 cleavages) and thimet oligopeptidase (116) have the most. There are 43 peptidases with cleavages in a hundred or more proteins, including cleavages in peptides that can be mapped to protein sequences in the UniProt database. Once again, because of its use in proteomics, more proteins are known that are susceptible to trypsin (3188) than any other peptidase. The methods by which cleavages were identified include mass spectroscopy (40,729 cleavages), N-terminal sequencing (8284), from knowing the consensus cleavage site (1609), amino acid analysis (205) and site-directed mutagenesis (178).

### 3.3 Substrate specificity

Table 4 shows the specificity of peptidases based on the occurrence of amino acids in the binding pockets P4–P4′. The preferences for each peptidase are defined in words so that the table represents a classification by specificity and homology. This also allows classification by amino acid type (acidic, basic, aliphatic, aromatic, *etc*) which would not be possible if logos were shown. The MEROPS identifier for each peptidase is a link to the peptidase summary page on the MEROPS website where the specificity logo is displayed. Caution should be exercised where these preferences are derived from fewer than 40 substrates. The number of peptidases for which the cleavage data are considered reliable (highlighted in green) is 319. The number for which the data are less reliable (yellow highlighting) is 123, and the number for which the data should be treated with caution (red highlighting) is 68. The vast majority of peptidases with a low reliability score are those showing apparent specificity in more than three binding pockets. It should be noted, however, that the reliability score will be affected by the number of binding pockets in which specificity is shown: this is apparent for the deubiquitinating hydrolases where the substrates positions P4–P1 are occupied in physiological substrates by the highly conserved C-terminus of ubiquitin (Leu–Arg–Gly–Gly). Because proteins substrates are unlikely to be homologous, a peptidase with many protein substrates will have a large reliability score. For a peptidase with cleavages in predominantly synthetic substrates, the reliability score will be low, because synthetic substrates tend to be similar with only the blocking and reporter groups differing. The peptidases with the highest reliability scores (96%) are asclepain A (C01.008); the snake venom enzyme jerdohagin (M12.216) and bpr peptidase from *Dichelobacter nodosus* (S08.022). In all three examples, protein cleavages are from the insulin B-chain [35], [36], [37]. The peptidase for which the reliability score is lowest (12%) is polyglycine endopeptidase (U9G.075): most known cleavages are from glycine-rich regions in endochitinases [38].

The table shows where one or two amino acids predominate in any binding pocket, or where a defined group of amino acids predominate. Also shown are one or two amino acids that are unknown in any substrate binding pocket where no preference is known to exist. Because some amino acids are rare or rarely encountered around a cleavage site, for example cysteine and tryptophan, these negative preferences are only shown when 200 or more cleavages are known for the peptidase. Where a binding pocket does not exist, for example P4–P2 for an aminopeptidase or P2′–P4′ for a carboxypeptidase, these are shown with a grey background. Please note that for some exopeptidases the name indicates a preference and not a strict specificity. For example, bacterial-type alanyl aminopeptidase (M01.005) has also been shown to act as an endopeptidase [39]. DmpA aminopeptidase (P01.001) also acts as an endopeptidase, processing its own precursor [40]. For many aminopeptidases, only cleavages in synthetic substrates are known and these frequently lack residues in positions P2′ to P4′. These apparent absences in binding pockets S2′–S4′ are not shown in Table 4 because they would be misleading.

The majority of peptidases for which specificity can be inferred (107 endopeptidases and 43 exopeptidases) show a preference in a single substrate binding pocket. Most of these peptidases show a preference in the S1 pocket, but there is a preference in each binding pocket S4–S4′ for at least one endopeptidase. There is an overwhelming bias for basic residues in P1, which probably reflects the large number of characterized peptidases in family S1. Exopeptidases, perhaps unsurprisingly, show preference only in pockets P1 and P1′. The TET aminopeptidase is the only exopeptidase to show a preference beyond the residues either side of the cleavage site.

Several peptidases show a preference in more than one binding pocket. There are 79 peptidases with a preference in two binding pockets, 50 peptidases with a preference in three pockets and 29 peptidases with a preference in four pockets. The table also includes examples of peptidases with preferences in more binding pockets, but as with the case for thimet oligopeptidase (see below) these apparent preferences are more likely to reflect the design of the substrates tested rather than true specificities.

Despite the large number of cleavages in substrates, the specificity of some peptidases cannot be explained in terms of preferences in binding pockets. Table 4 shows the 53 peptidases with more than forty substrate cleavages for which no preference is shown. This list includes some well-known and well-characterized peptidases such as cathepsin G, for which even a large proteomics study was unable to show any preference except that lysine is not acceptable in P4 [27].

Table 4 also shows that peptidases with similar specificities can be unrelated to one another (members of different families or even catalytic types), for example gingipain K and bacterial lysyl endopeptidase both have a preference for Lys in P1, yet the former is a cysteine peptidase and the latter a serine peptidase. There are also examples where specificity differs markedly from peptidases with homologous sequences, with examples from family S1 being the most well known in which chymotrypsin A has a preference for aromatic residues in P1, trypsin 1 for basic residues, elastase-2 for aliphatic residues and granzyme B for aspartic acid. It is more unusual for peptidases within a family to utilise different substrate binding pockets, but an example is granzyme B which also shows a preference for an aliphatic residue in P4. A family may include exo-as well as endopeptidases, for example family S9 includes prolyl oligopeptidase and dipeptidyl-peptidase IV, although both have a preference for Pro in P1. These facts emphasize that peptidases cannot be classified to the protein species by specificity or sequence similarity alone, but that both specificity and homology should be taken into consideration.

There are many peptidases for which the number of physiological substrates is limited. These include proteins that process themselves to remove a propeptide or to expose a N-terminal nucleophile and then cease to function as peptidases. Examples include the amidophosphoribosyltransferase precursor (C44.001) [41] and the penicillin V acylase precursor (C59.001) [42], for both of which the only known cleavage is to expose a new N-terminal nucleophilic cysteine. Human peptidases such as renin (A01.007) have few natural substrates but the number of cleavages is boosted by synthetic substrates and peptides.

### 3.4 Displays on the MEROPS website

A variety of displays have been implemented on the MEROPS website to aid understanding of peptidase specificity. The displays include the following.

**Fig. 2:**
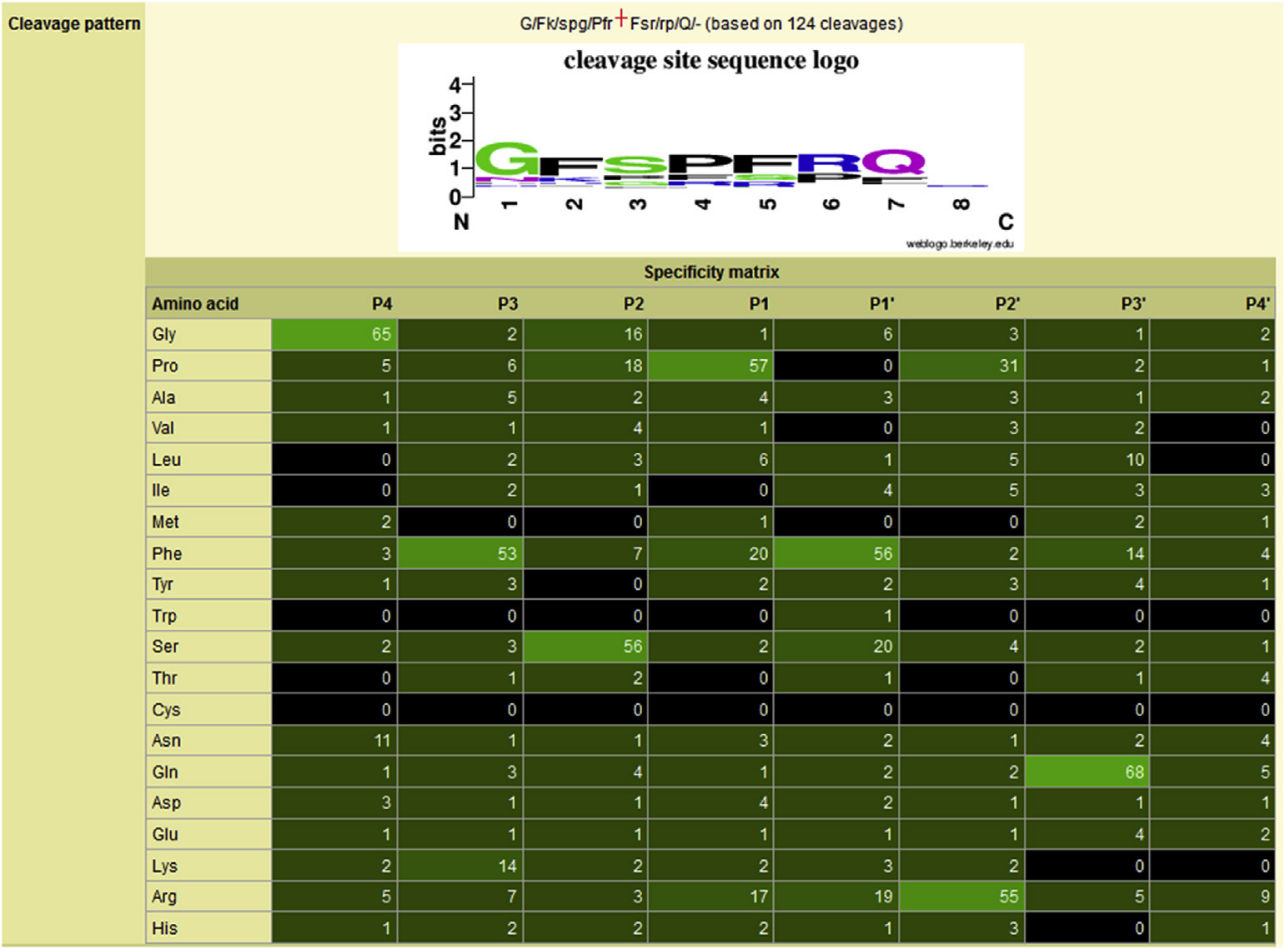
**Example of a specificity logo and distribution matrix**. The specificity logo and distribution matrix are shown for thimet oligopeptidase. In the logo, the taller the character the greater the preference in substrate binding pockets S4 to S4′ (numbered as 1 to 8 on the X axis). In the specificity matrix the number of times an amino acid occurs in the residue range P4 to P4′ in substrates is shown. The brighter the green highlighting, the greater the preference for an amino acid in that position. An amino acid that has not been observed to occupy a specific binding pocket is shown as white text on a black background. Amino acids are ordered so that amino acids with similar properties are grouped together.

In each peptidase summary, where ten or more substrate cleavages are known, a logo (generated by the Weblogo software, http://weblogo.berkeley.edu/) and a specificity matrix are shown. Fig. 2 shows the logo and specificity matrix for thimet oligopeptidase. In the logo, amino acid residues are shown as single-letter abbreviations, and the greater the height of the letters the greater the preference for the amino acid in that binding pocket [43]. The binding pockets S4–S4′ are numbered along the X axis as 1–8. In the specificity matrix, amino acids are shown in three-letter notation, and the number of times each occurs within the range P4–P4′ for a substrate is shown. The occurrence of each amino acid in each binding pocket is calculated as a percentage of total cleavages, and a different shade of green is used for each tenth percentile. The brighter green the background highlighting, the greater the preference for that amino acid in the corresponding substrate binding pocket. If an amino acid has not been observed in any binding pocket, then it is shown as white letters on a black background. Obviously, with some amino acids that occur less frequently (Cys and Trp, for example), the number of cleavages should be taken into consideration before any conclusions are drawn about negative preferences by the peptidase.

The example in Fig. 2 is based on 125 cleavages. However, 80 of these cleavages are derived from one reference [44] in which the substrates based on Abz-GFSPFRQ-EDDnp were synthesized to help distinguish thimet oligopeptidase from its close relative neurolysin. It is immediately obvious that the preferences shown in binding pockets S4–S3′ (Gly, Phe, Ser, Pro, Phe, Arg, Gln) reflect the residues that were kept constant during the residue-scanning experiments. This example serves as a cautionary note to show that apparent preferences may reflect the methodology employed rather than represent the true specificity of the peptidase.

**Fig. 3:**
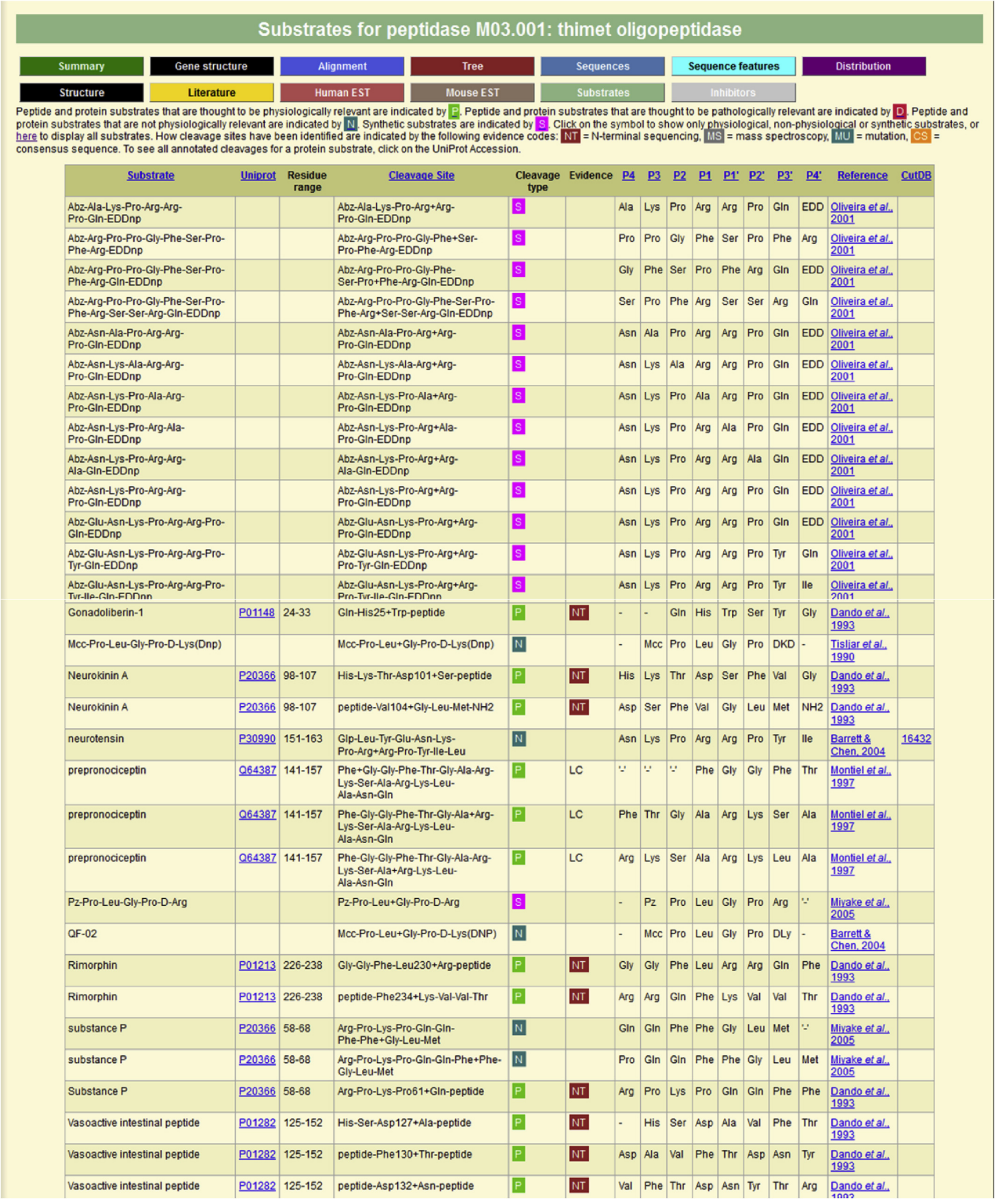
**Example of a substrate page**. Part of the substrates page for thimet oligopeptidase is shown. For each substrate the following are shown: name; a cross-reference and link to the entry in the UniProt database where appropriate; the residue range of the substrate as used in the experiment with reference to the numbering in the UniProt entry; a description of the cleavage where the scissile bond is represented by the symbol ‘+’; whether the cleavage is physiological, non-physiological, pathological or in a synthetic substrate; the evidence by which the cleavage site was determined; the residues occupying residues P4 to P4′ in the substrate; the source reference; and a cross-reference and link to the CutDB database [14]. By default substrates are listed alphabetically, but the order can be changed by clicking the column heading. It is possible to filter the results for physiological, nonphysiological, pathological or cleavages in synthetic substrates by clicking on the appropriate letter in the table legend.

For each peptidase where substrate cleavages are known there is a page dedicated to listing the substrates and the known cleavage positions. Fig. 3 shows sections of the table of substrates for thimet oligopeptidase. For each cleavage, the substrate name is given, the UniProt accession if the substrate is from a naturally occurring protein, the residue range of the substrate with respect to the Uniprot entry, the cleavage site, the nature of the substrate (“cleavage type”), the evidence for the cleavage position (if known), a reference and a cross-reference to the CutDB database [14].

**Fig. 4:**
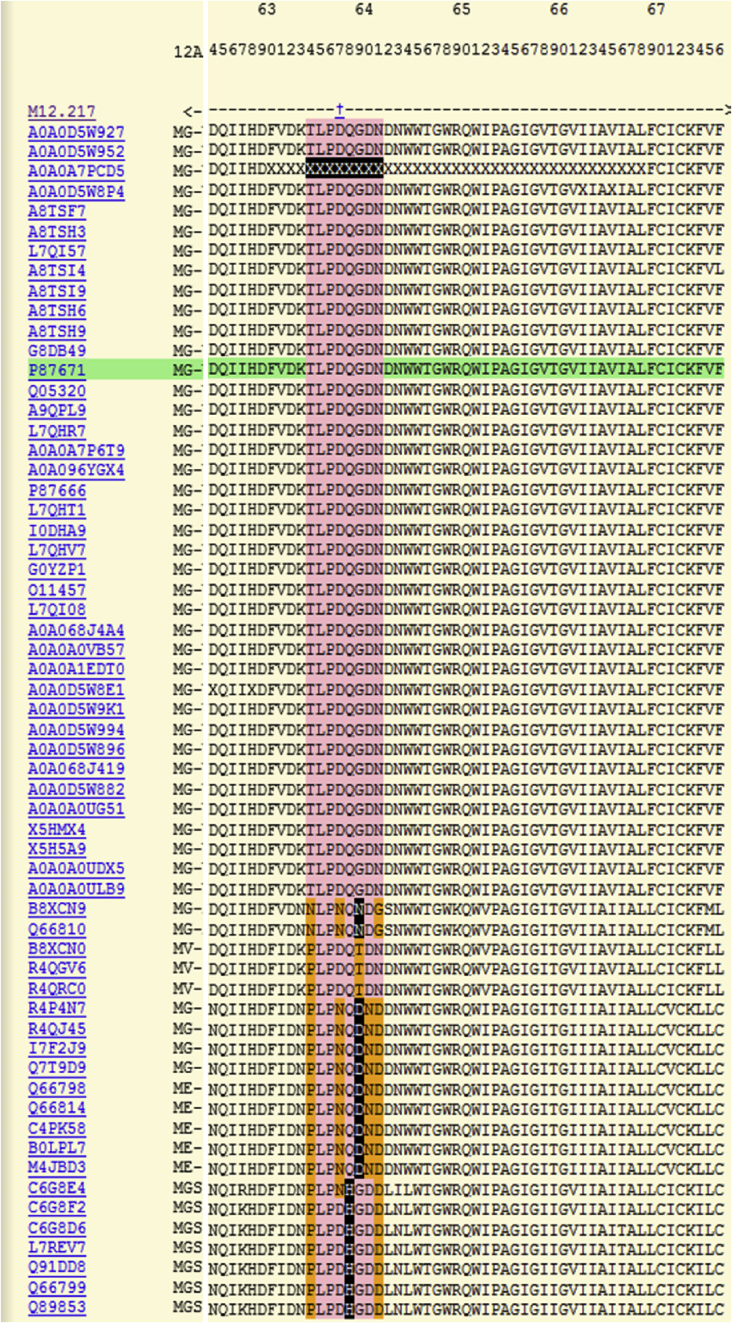
**Example of a substrate alignment**. Part of the alignment for orthologues of the Ebola virus envelope glycoprotein is shown, with the known cleavage of the glycoprotein from the Zaire strain (UniProt P87671) by ADAM17 (MEROPS ID M12.217) at residue 637 highlighted in green. Residues in the range P4–P4′ are highlighted in pink if they are identical to that from the Zaire strain; substituted residues are highlighted in orange if the amino acid from another ADAM17 substrate is known to occupy the same binding pocket; and substituted residues are shown as white on black if the amino acid is not known to occupy the same binding pocket from any ADAM17 substrate.

There are also displays for protein substrates. One important question when considering whether a particular cleavage is physiologically relevant is whether the cleavage site is conserved in close homologues. A cleavage site that is not conserved is unlikely to be physiologically relevant, although it may be pathologically relevant in the species in which it occurs. Conservation should be in terms of which residues are accepted in the binding pockets of the peptidase and not just sequence conservation in the substrate orthologues. This is only possible if a large number of substrate cleavages are known for the peptidase in question. Fig. 4 shows part of the alignment for orthologues of the Ebola virus envelope glycoprotein. The known cleavage is from the Zaire strain (UniProt P87671) and cleavage is by ADAM17 (MEROPS ID M12.217) at residue 637; the cleavage results in shedding of the ectodomain which circulates in the blood of the patient and may interfere with antibodies thus helping to prolong infection [45]. The alignment is dynamically generated from the sequences clustered in the UniRef50 database entry that contains this sequence. The UniRef50 database entry contains sequences that share 50% or more sequence identity [46]. Alignments are generated using MUSCLE [47]. The sequence containing the known cleavage site is highlighted in green. Residues in the range P4–P4′ are highlighted in pink if they are identical to that from the Zaire strain; substituted residues are highlighted in orange if the amino acid from another ADAM17 substrate is known to occupy the same binding pocket; and substituted residues are shown as white on black if the amino acid is not known to occupy the same binding pocket from any ADAM17 substrate (“unacceptable replacements”).

In this example, in one sequence the residues around the cleavage site have not been determined and are replaced by Xs, and in sequences from a number of strains of the virus the P1′ residue is replaced by His and the P2′ residue by Asp or Asn, which have not been observed in any of the 59 other substrates for ADAM17. The possible conclusions are: 1) ADAM17 is not the physiological peptidase that performs this cleavage, 2) some Ebola virus envelope glycoproteins are processed by a different peptidase, 3) the specificity of ADAM17 has not been fully explored and His is permissible in P1′ and Asp or Asn in P2′, or 4) it doesn't matter if the cleavage is inefficient in some cases provided some processing occurs.

### 3.5 A service to test conservation of cleavage sites

The production of this cleavage collection has allowed the installation of a service on the MEROPS website to help researchers assess whether or not a particular cleavage is physiologically relevant. Following on from the display described above, the philosophy behind this service is that a cleavage site in a protein substrate is most likely to be physiologically relevant if it is conserved. The user can submit a list of cleavage sites in a file the structure of which is shown in Table 5. This should be a text file created with software such as Notepad and not a document file created with a word processing package such as Word. The data required per line are: MEROPS identifier of the peptidase performing the cleavage, the UniProt accession for the substrate protein, and the position of the residue occupying the S1 binding pocket (the residue after which cleavage occurs) taken from the full coding sequence in the UniProt entry. For each line, an alignment is generated from the sequences in the UniRef50 entry containing the UniProt accession of the substrate protein, and the number of unacceptable replacements is counted. A file is generated and is E-mailed to the user. An example of a results file is shown in Table 6. For each line in the table the following are included: the MEROPS identifier of the peptidase; the total number of cleavages known for that peptidase; the UniProt accession of the substrate protein; the number of homologues aligned from the UniRef50 entry; the cleavage position; the number of unacceptable replacements at P4, P3, P2, P1, P1′, P2′, P3′ and P4′; and a URL to display the substrate alignment at the MEROPS website (to conserve space, the URL column is not shown in Table 6). The number of known substrate cleavages for the peptidase is returned because this will help the user assess the reliability of the analysis: the more cleavages the more reliable the analysis. Similarly, the number of sequences in the alignment will help the user assess the results: if the alignment contains many sequences and the cleavage site is well conserved, then the likelihood that the cleavage site is physiologically relevant is greater. If on the other hand the cleavage site is not conserved, and there are many sequences in the alignment, then it is much more likely that the cleavage is not physiologically relevant.

If the number of unacceptable replacements in positions P4–P4′ is zero, then the cleavage site is extremely well conserved and is likely to represent a physiological cleavage by the peptidase concerned. If the number of unacceptable replacements is small, then the cleavage may still be physiological. The most common explanation is that the UniRef50 entry on which the alignment is based contains two or more very closely related paralogues, one of which is a physiological substrate, but the other is not. A second possibility is that the sequences in the UniRef50 entry represent species variants of only one protein, but one or more is derived from genomic sequencing and is either a fragment (perhaps because the initiating methionine has been misidentified or an anomalous frame-shift has been introduced which truncates the C-terminus), or is missing the exon which codes for all or part of the cleavage site. The user is advised to consult the alignment via the URL provided to check. If the number of unaccepted replacements is high, then the cleavage site is not conserved and the cleavage is unlikely to be physiologically significant. However, the user should check the number of cleavages for the peptidase: if this is less than 40, then it is possible that there is not enough variation amongst the known cleavage site sequences to account for the substitutions that have occurred. It is possible that many homologues in the alignment have the same replacement which has not been observed in any substrate for the peptidase, simply because not enough substrates have been found.

The user should also be aware that if there is a high number of unacceptable replacements in one position, this requires further investigation, because substitution to a rare amino acid may have taken place. For a peptidase with no preference in a binding pocket, for example S3′, almost any amino acid can occupy the P3′ position. In the alignment of substrate homologues, the count of unacceptable replacements is therefore likely to be small. However, if no substrate is known with cysteine in P3′, for example, yet many homologues of a known substrate have a replacement cysteine in P3′, then the unacceptable replacement count will be high. In such an example, it is simply not known if cysteine is acceptable in P3′, so it is impossible to say if the cleavage is physiological or not. The user should check the alignment for such a replacement.

I would like to reiterate that a cleavage site that is not conserved could still be important pathologically. The user is advised to check the preferences for the peptidase on the peptidase summary page in MEROPS to see if, for example, a large number of mismatches in P4′ is relevant.

This service is known as “Analyse Substrates” and is accessed from the main menu on the website. It is particularly useful for proteomics studies in which hundreds of potential physiological substrates are found. The limitations on the service are that the maximum number of cleavages per file uploaded is 5000 and the maximum file size is 10 Mbytes.

## 4 Conclusions

Cleavages in substrates (proteins, peptides and synthetic substrates) by proteolytic enzymes have been collected from the literature. In total, 66,615 cleavages have been annotated for 1700 different peptidases (69% of the 2457 different peptidases so far identified). The number of cleavages per peptidase varies greatly, from zero to 13,770 for trypsin 1. Peptidases with the most known cleavages are derived from proteomics experiments.

Users should be aware of the biases that may exist in the substrate cleavage data for a peptidase. If the data are predominantly derived from amino acid scanning experiments in which all but one amino acid is changed in a variety of synthetic peptides, then the apparent specificity will be affected by the amino acids that are kept constant. Similarly, if the cleavages are in synthetic substrates, then the amino acid variety may be limited with changes only to blocking and reporter groups. To address this problem, a “reliability score” has been introduced, which is the average percentage difference in the residues P4–P4′ for all substrates for a peptidase. A high reliability score will indicate a higher variety in residues occupying substrate binding sites. However, this score is affected when several of the substrate binding pockets that confer specificity.

By analysing the residues that occupy the P4–P4′ substrate binding sites, it has been possible to categorize the specificity of some peptidases. A logo and specificity matrix is shown on the MEROPS website for each of the 556 peptidases (22.7%) with ten or more substrate cleavages. Of these, 107 endopeptidases and 43 exopeptidases show a preference in one substrate binding pocket, 79 peptidases show a preference in two binding pockets, 50 show a preference at three binding pockets, 29 show a preference at four binding pockets and 53 show no positive preference in any substrate binding site. However, it has been proposed that at least 40 cleavages in disparate proteins are required for specificity analysis to be meaningful, and only 163 peptidases (6.6%) fulfil this criterion.

Proteomics experiments in which cleavage sites are identified by mass spectroscopy provides the bulk of the substrate cleavage data. However, a common problem is that in order to extract the proteins from the sample, the natural, physical boundaries that separate peptidase from substrate may have been removed, and a large number of substrate cleavages are generated. While this is not a problem if no claims are made about the physiological relevance of the data and the suite of peptides are generated just to examine peptidase specificity, there are issues when it is claimed that the experiment will reveal physiological substrates. The biggest concern is that not all substrates will be physiological, but distinguishing these from artefactual and bystander substrates is difficult. A bioinformatics approach that has been adopted here is to consider whether the cleavage is conserved in orthologues of the substrate protein. For each cleavage site, the UniProt accession of the substrate protein is determined, as well as the UniRef50 cluster of sequences to which it belongs. All protein sequences from the UniRef50 cluster are then aligned, and the residues P4–P4′ for each cleavage site are examined for conservation. The number of replacements that are not found to occupy the same binding pocket in any of the substrates for the peptidase is counted. If this number is high, then the likelihood that the substrate is physiologically relevant is low. A server has been set up at the MEROPS website whereby the results from a proteomics experiment to study peptidase specificity can be uploaded, and the conservation around each cleavage site examined. Results are returned by E-mail.

## Figures and Tables

**Table 1:** Counts of different peptidases (peptidase species) by catalytic type.

	Aspartic	Glutamic	Metallo	Cysteine	Serine	Threonine	Mixed	Asparagine lyases	Unknown	Total
Sequenced and characterized	170	7	633	615	942	46	5	23	16	2457
Sequenced only	118	0	327	297	644	29	0	1	3	1419
Sequence not known	8	0	86	19	87	1	0	0	49	250
Non-peptidase homologues	4	0	103	52	145	26	0	0	0	330
Pseudogenes	24	0	5	21	17	3	0	0	0	70
Total	324	7	1154	1004	1835	105	5	24	68	4526

**Table 2:** Counts of substrates per catalytic type.

	Aspartic	Glutamic	Metallo	Cysteine	Serine	Threonine	Mixed	Asparagine lyases	Unknown	Total
Physiological	2780	7	4113	7311	5877	33	2	60	81	20,264
Pathological	266	0	345	700	34	0	0	0	4	1349
Non-physiological	2893	70	8762	3112	21,301	84	1	3	3	36,229
Synthetic	364	32	1569	1179	2547	42	37	0	51	5821
Theoretical	176	0	545	106	638	0	0	300	0	1765
Unclassified	77	0	554	194	314	26	1	0	21	1187
Total	6556	109	15,888	12,602	30,711	185	41	363	160	66,615

**Table 3:** Counts of peptidases with known substrate cleavages by catalytic type.

	Aspartic	Glutamic	Metallo	Cysteine	Serine	Threonine	Mixed	Asparagine lyases	Unknown	Total
Sequenced and characterized	114	4	477	401	520	18	2	17	33	1586
Sequence not known	5	0	41	6	34	0	0	0	28	114
Total	119	4	518	407	554	18	2	17	61	1700

**Table 4:**
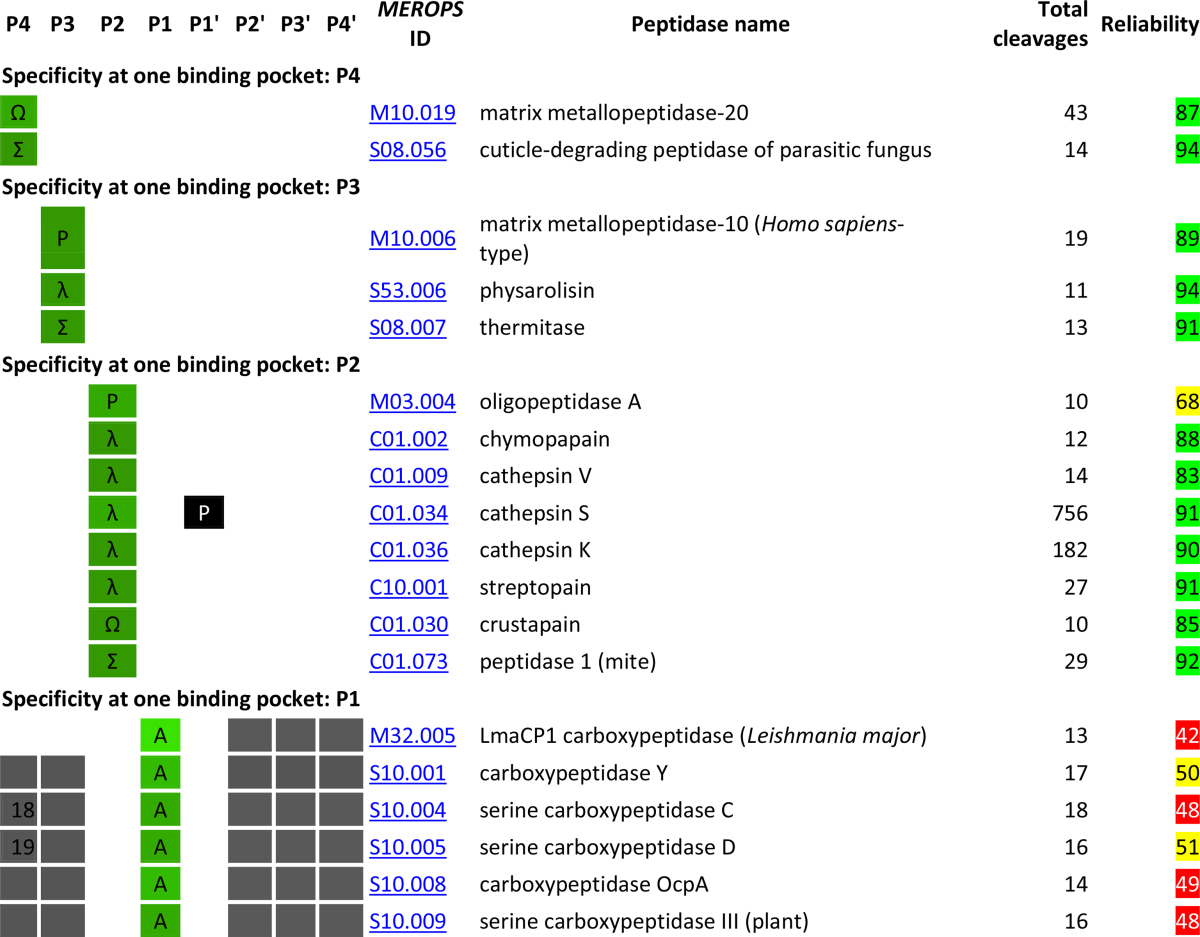

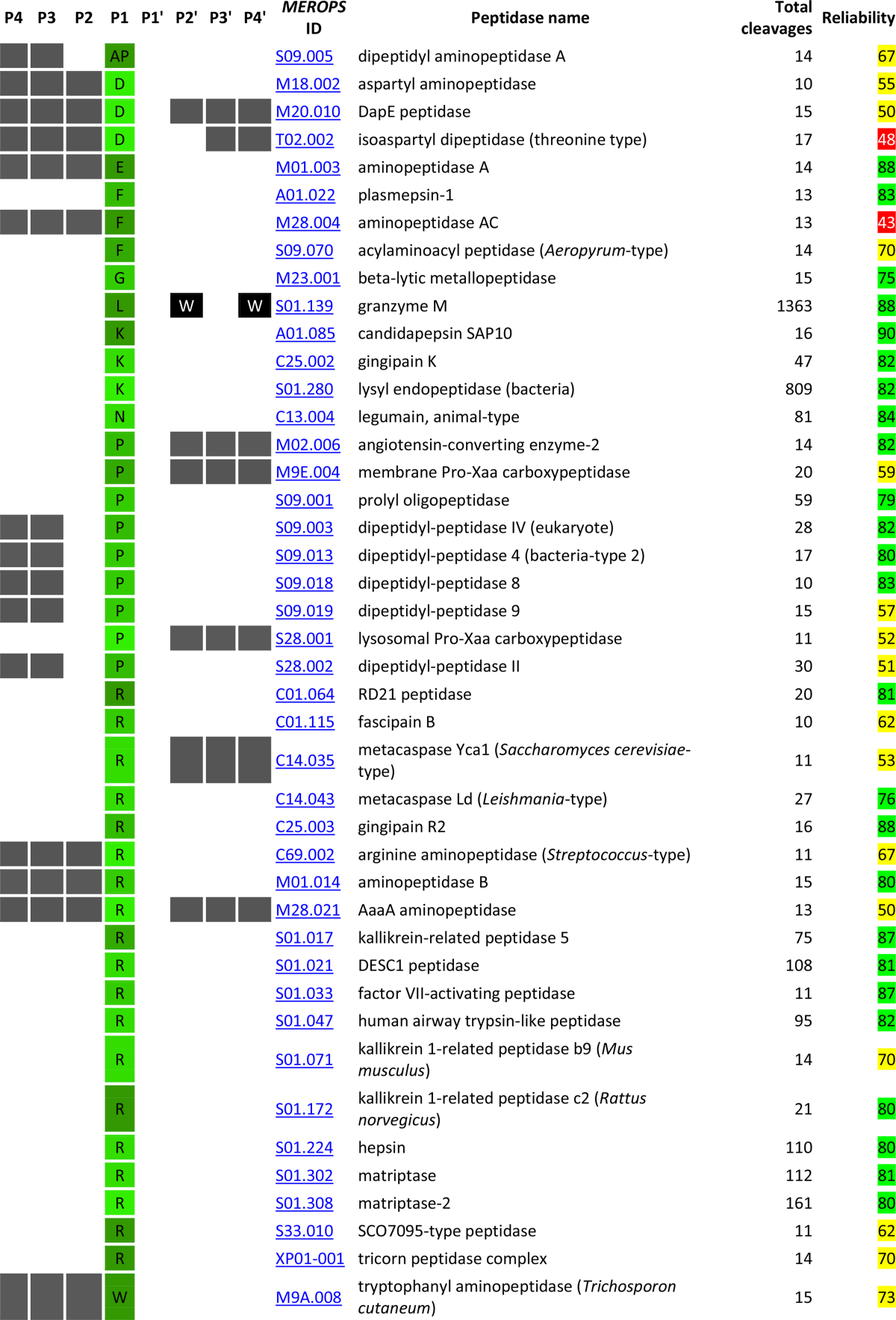

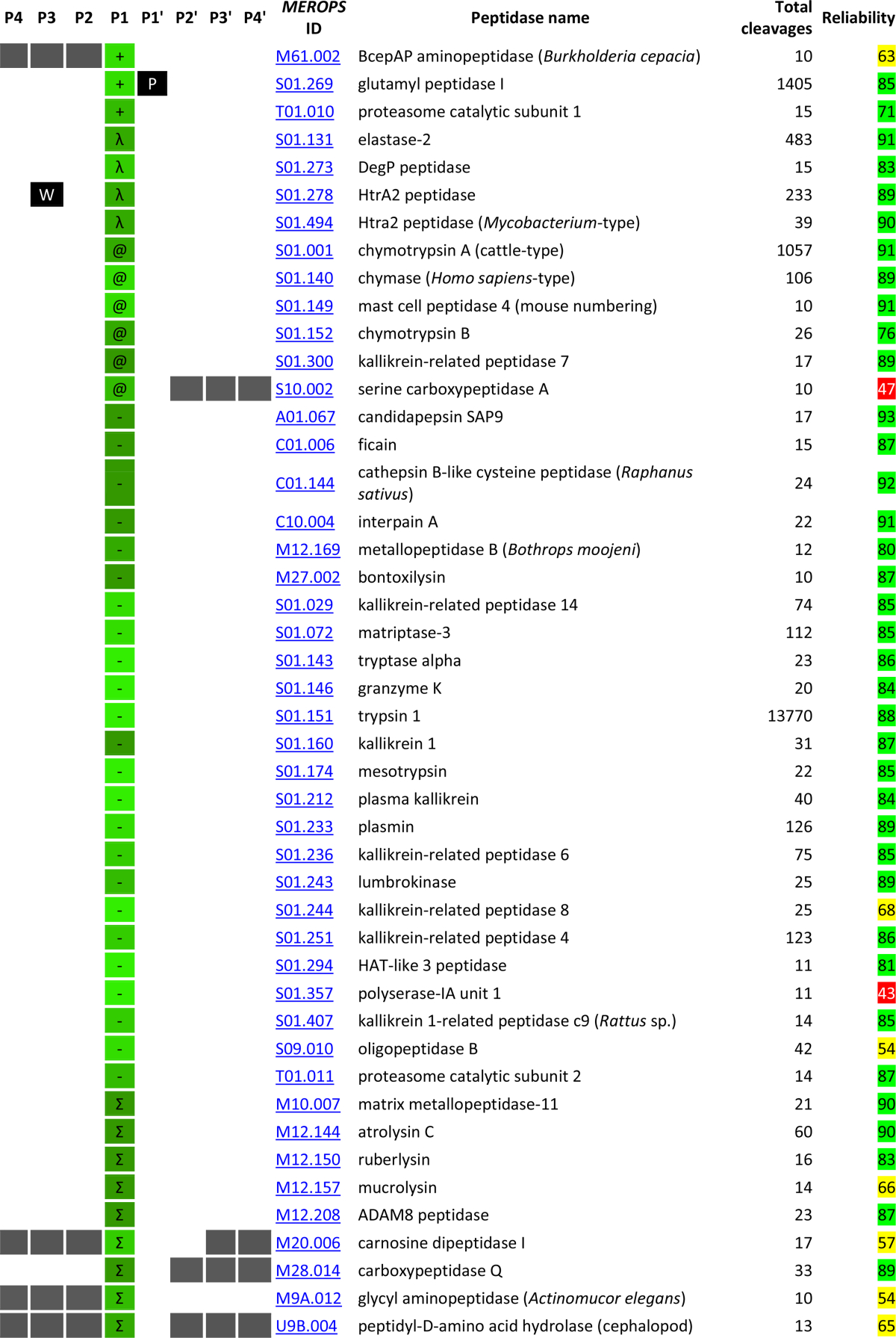

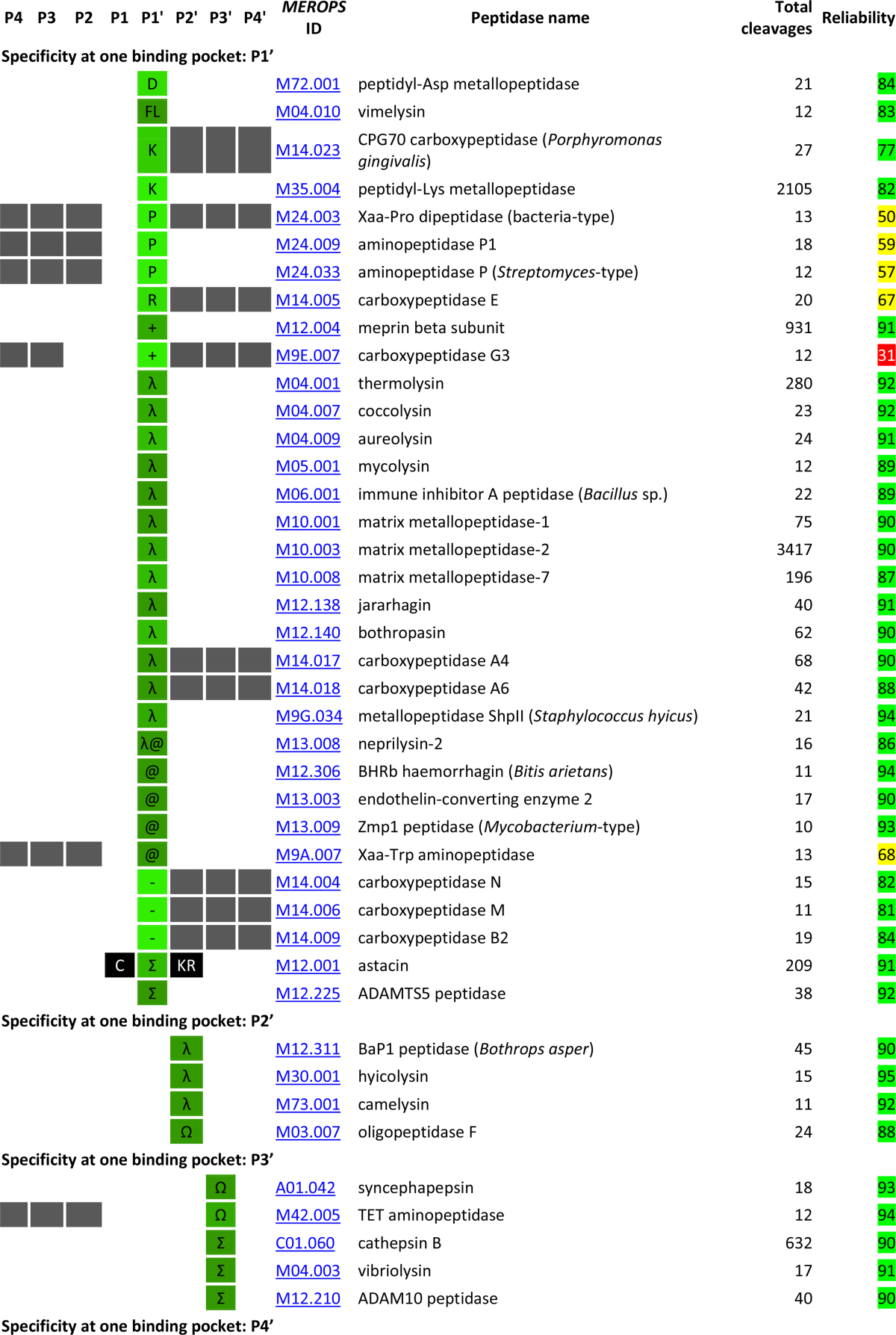

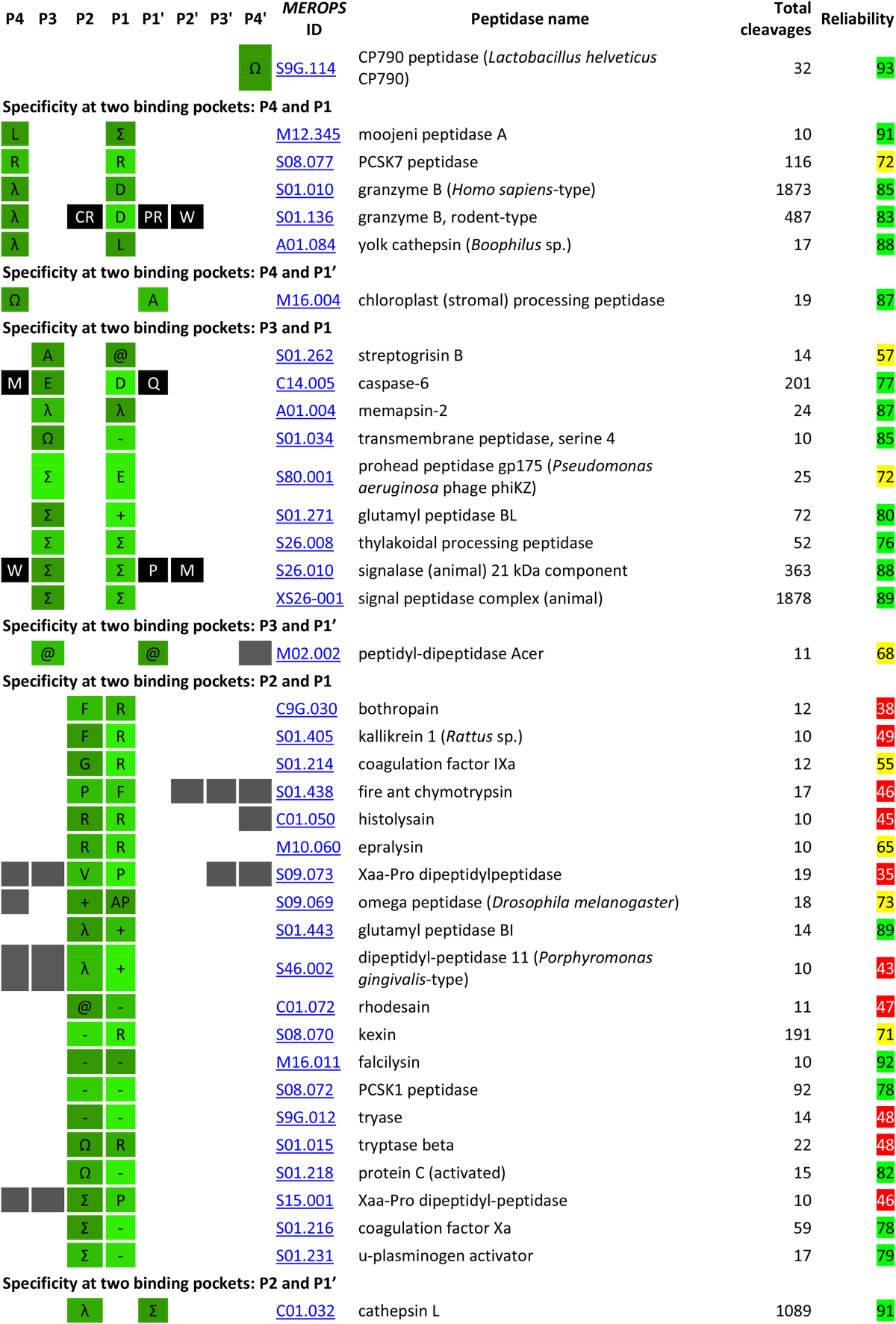

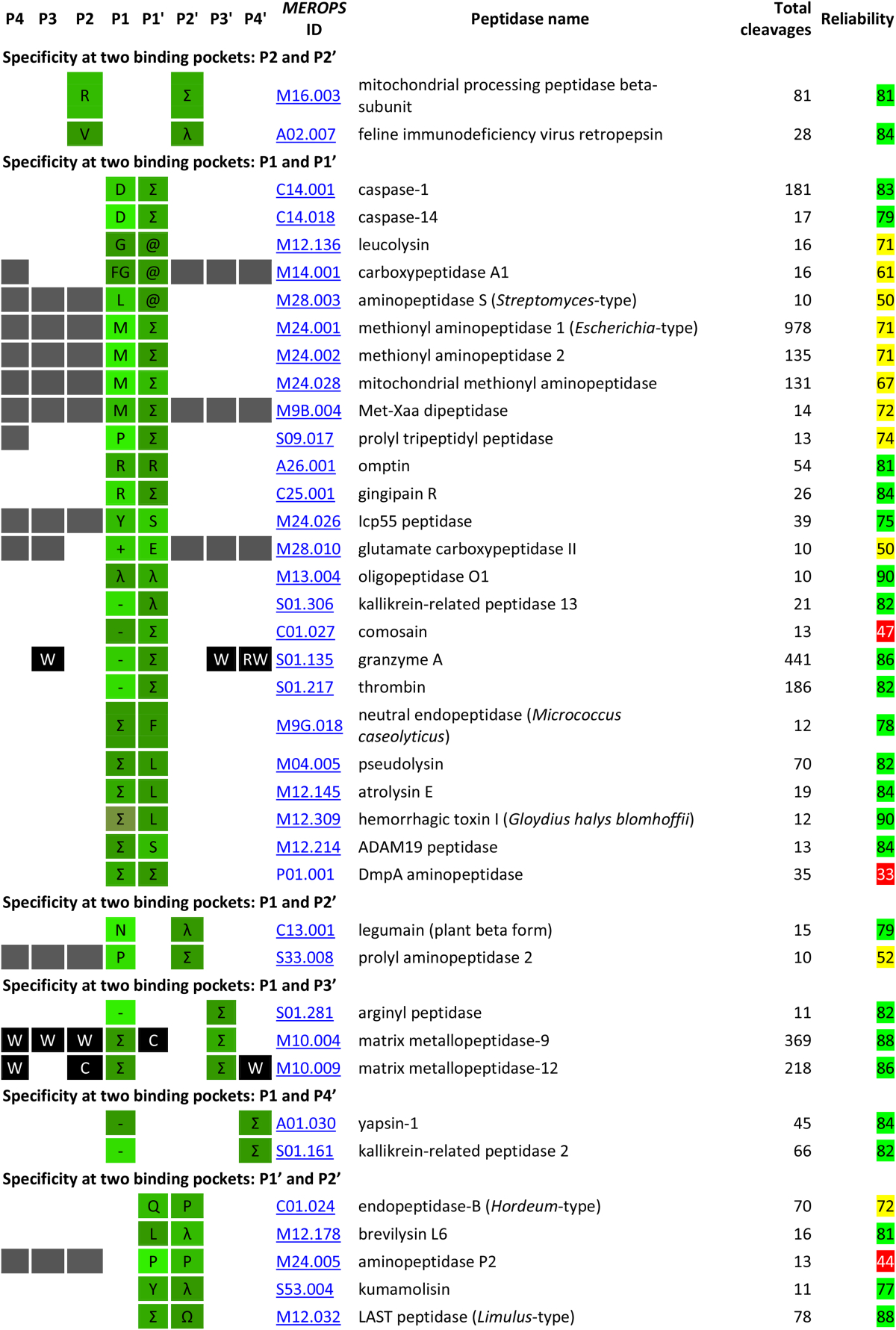

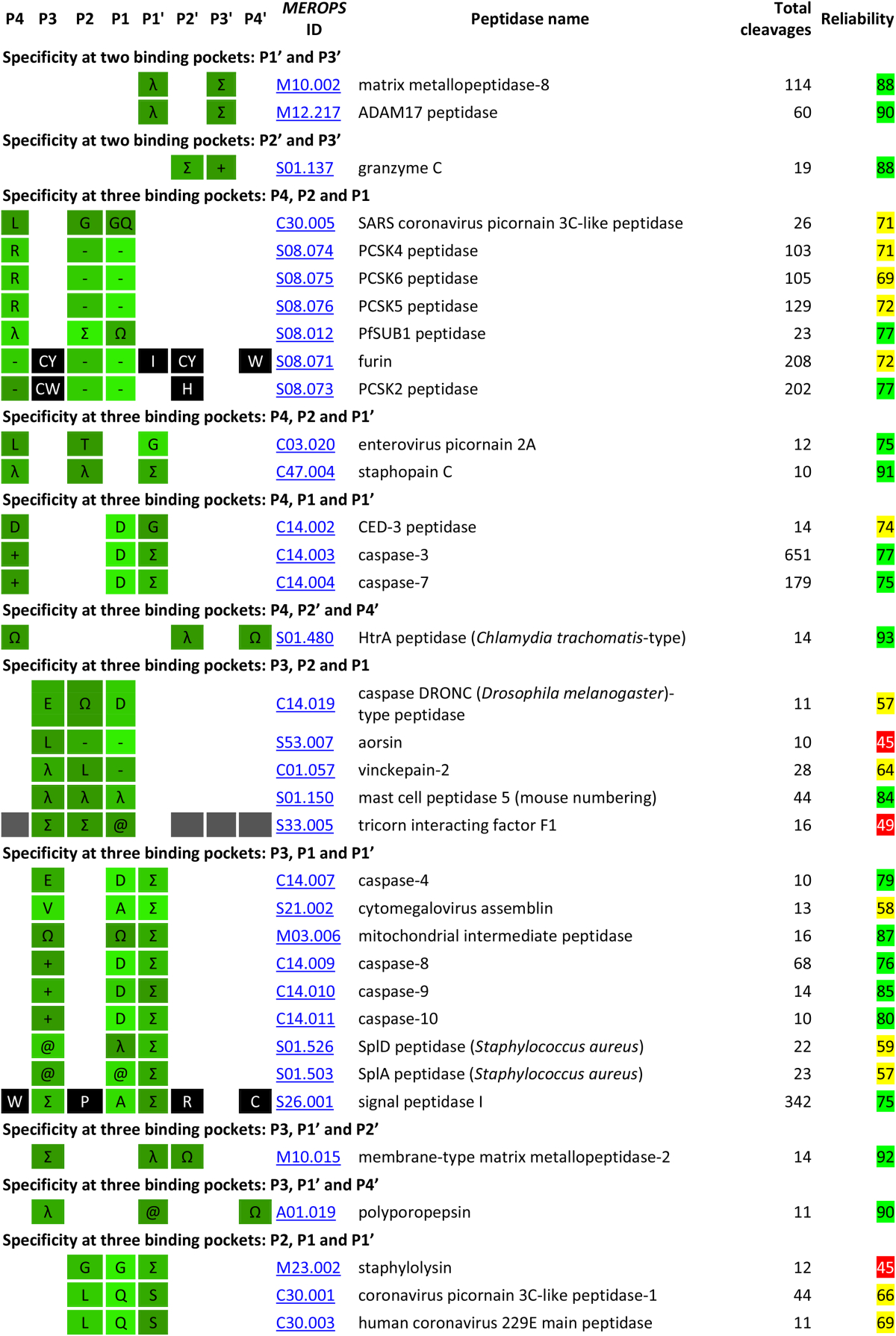

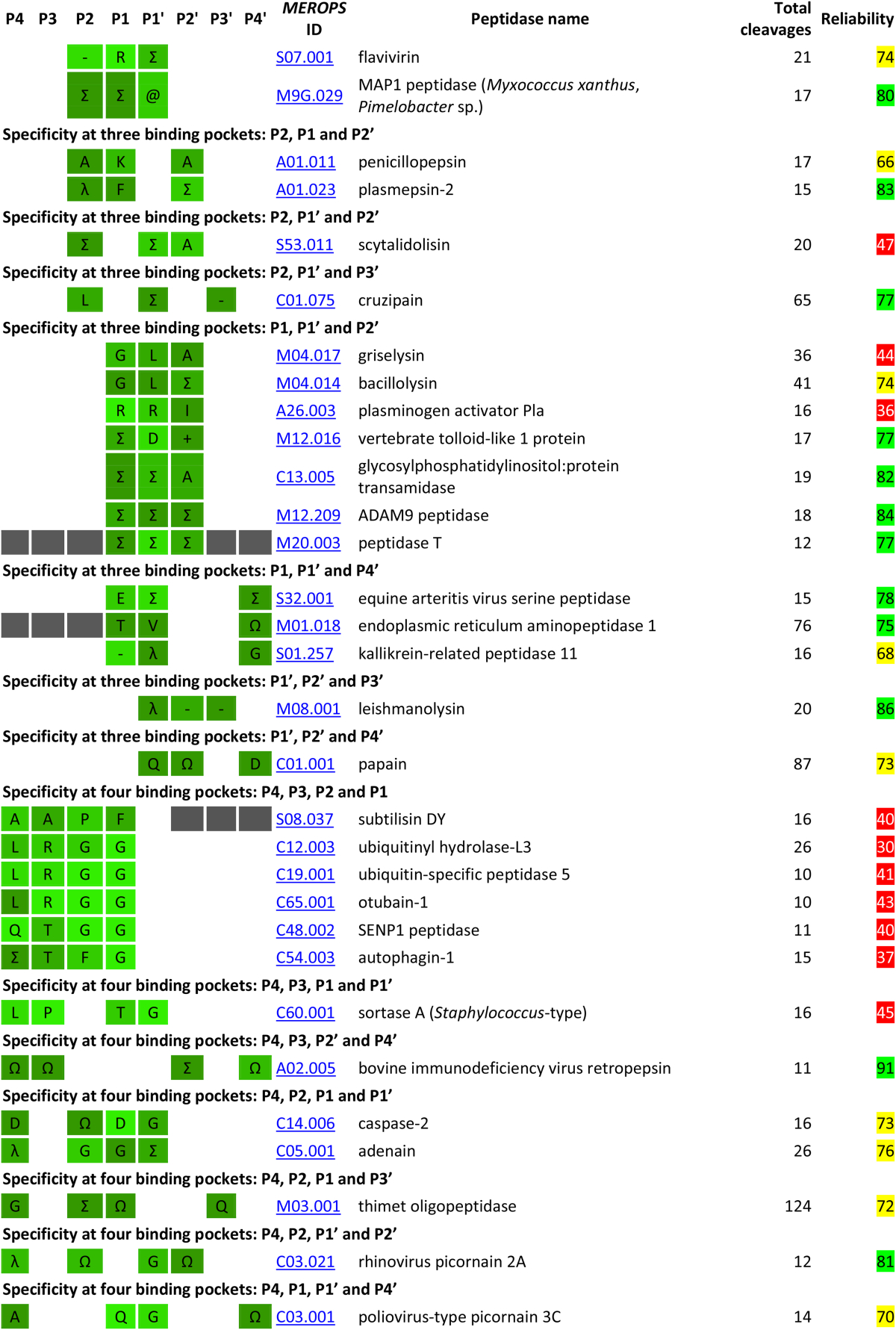

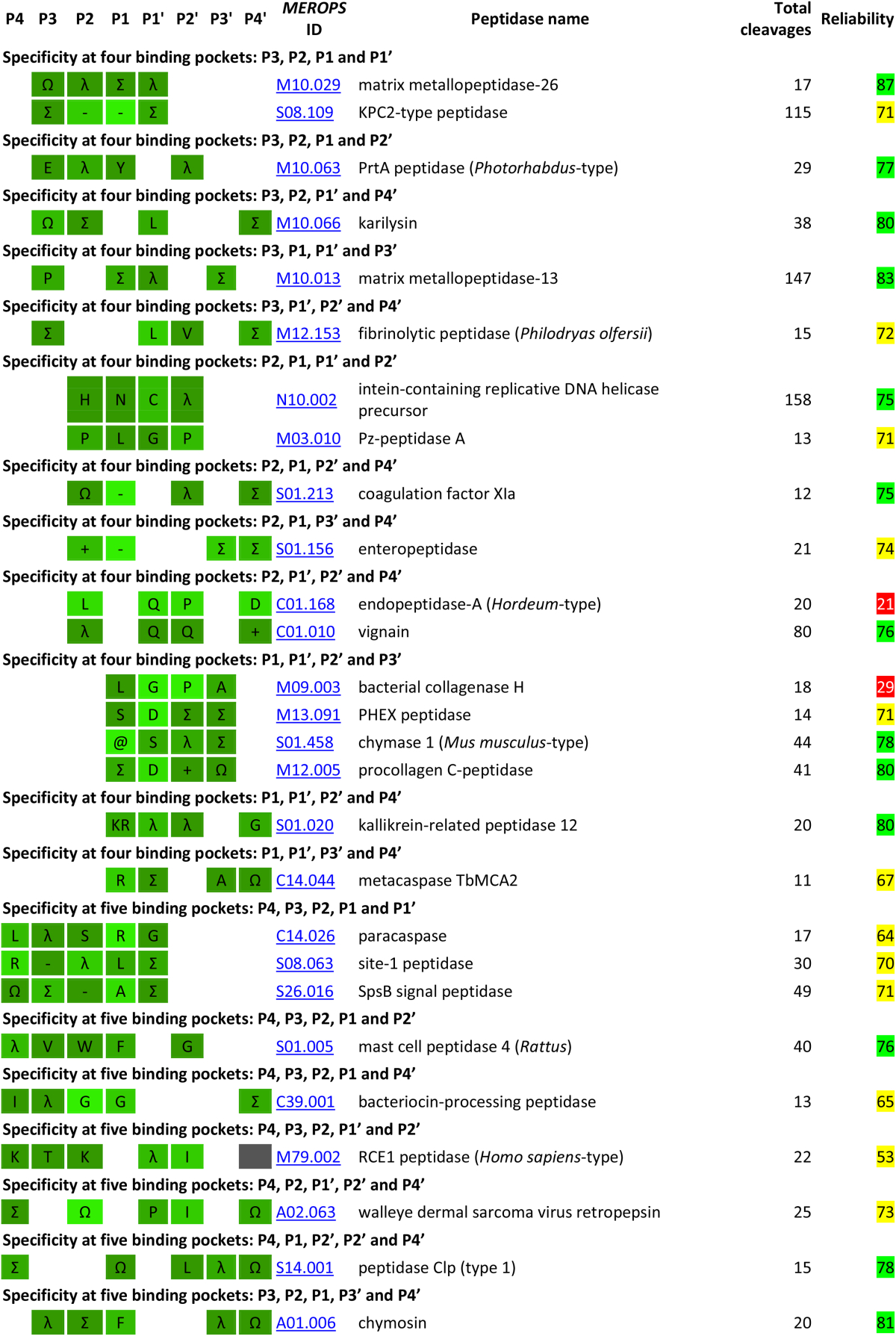

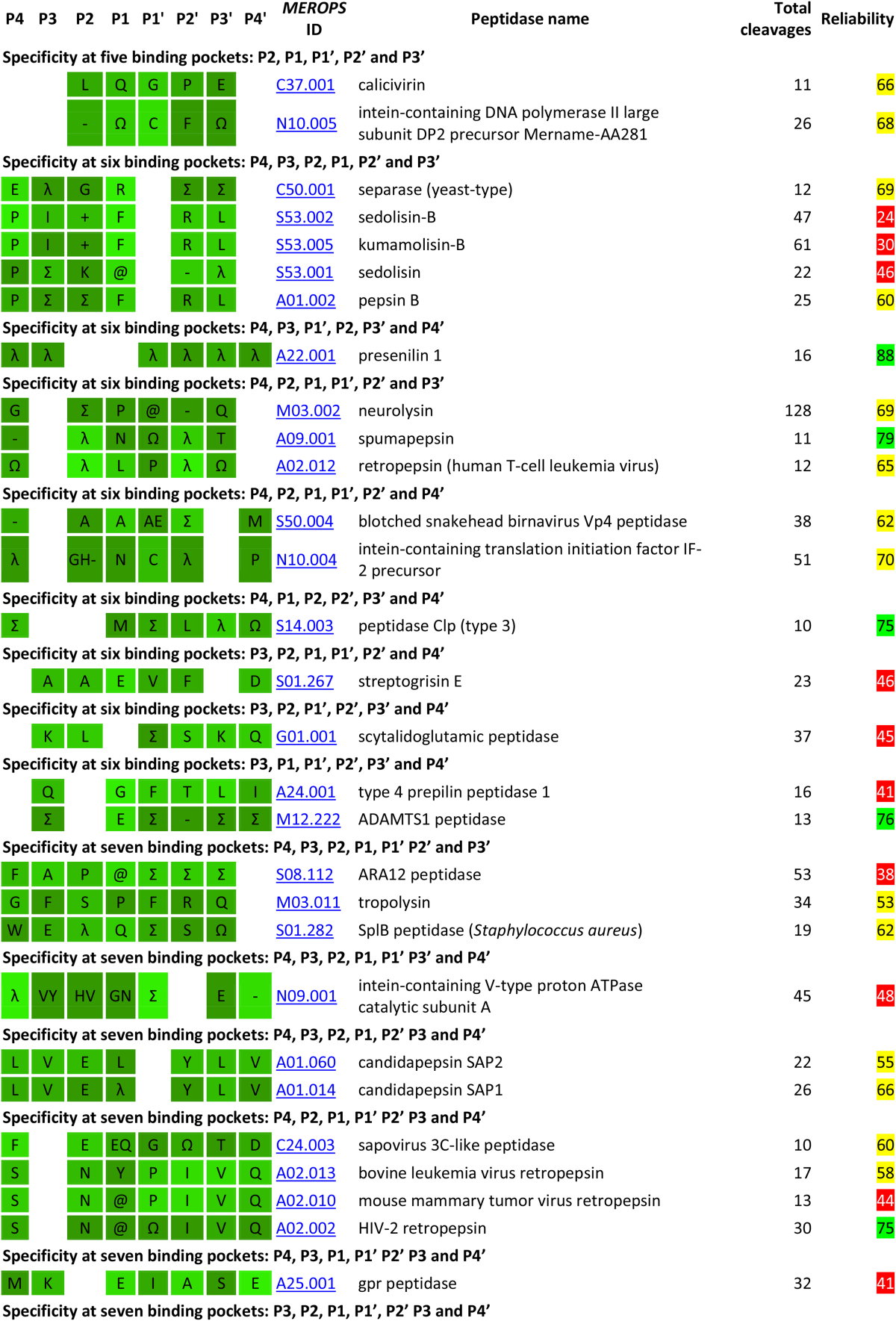

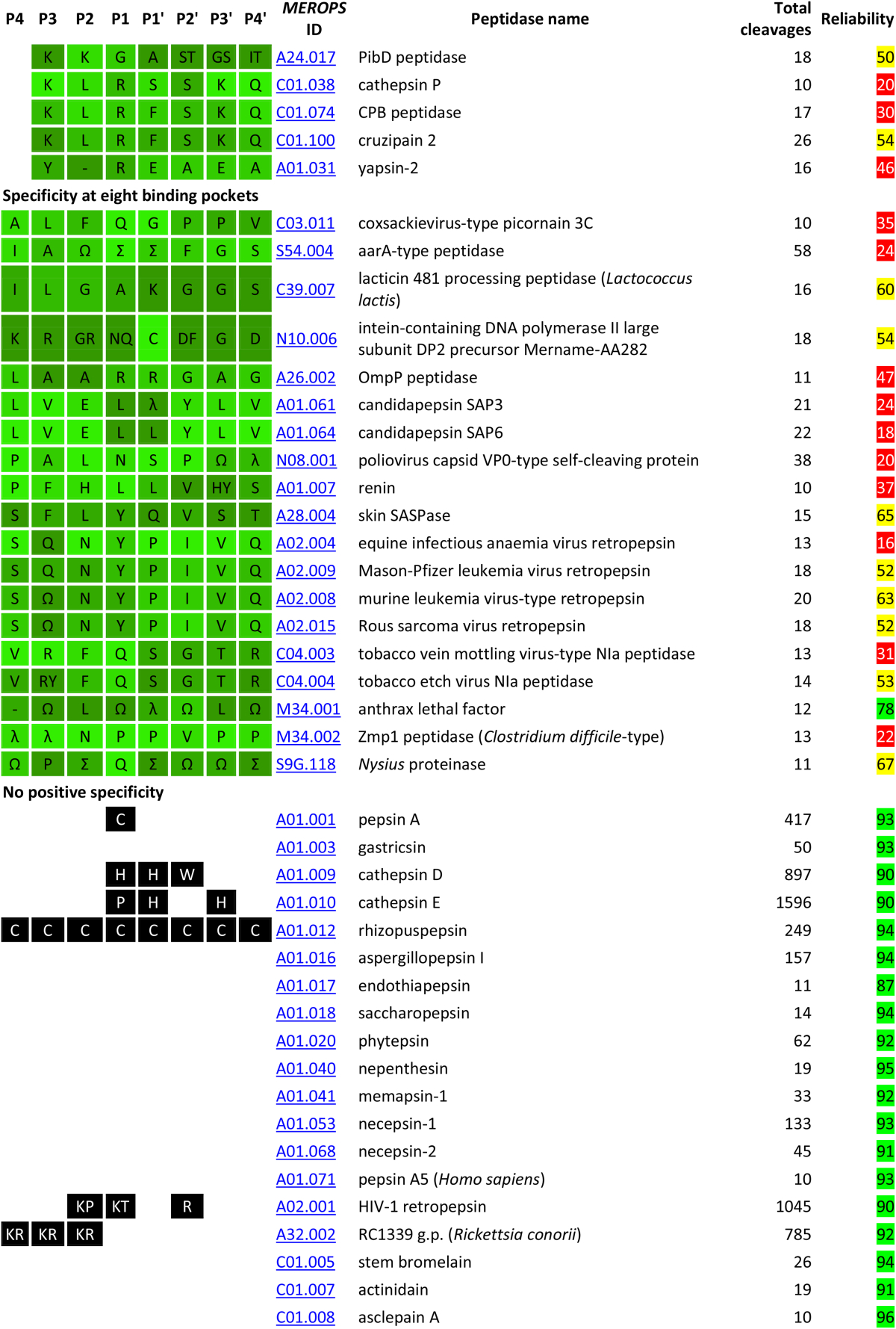

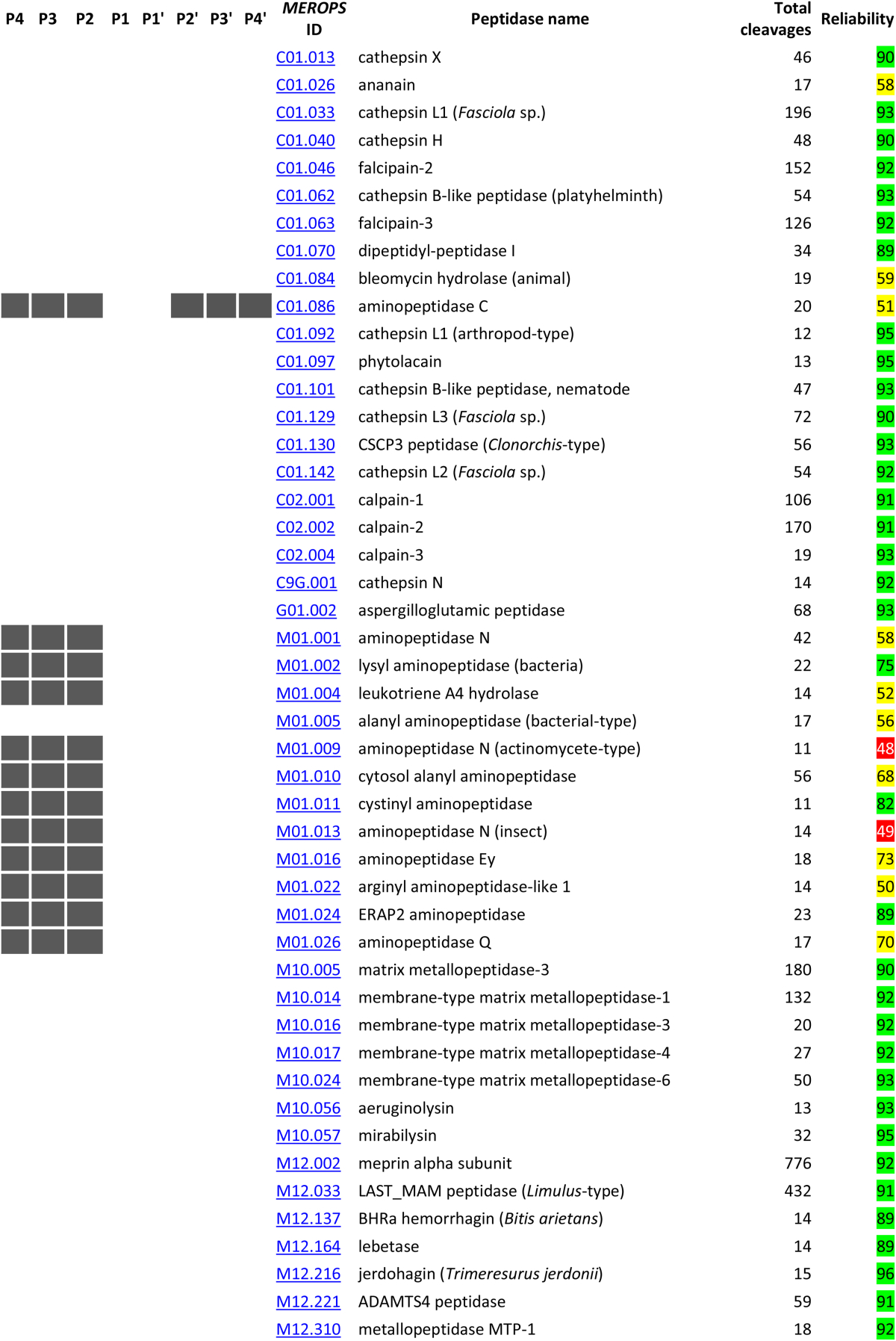

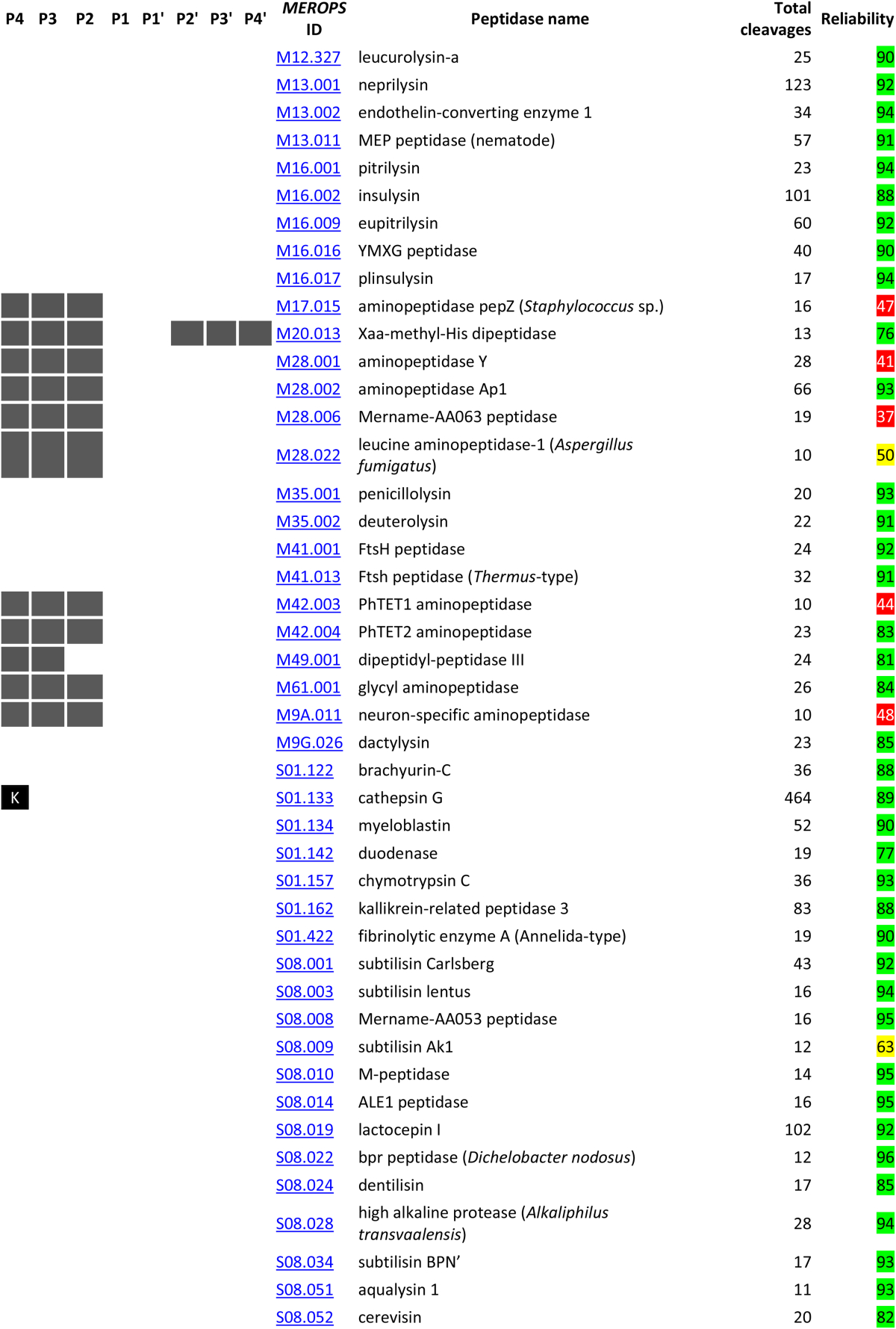

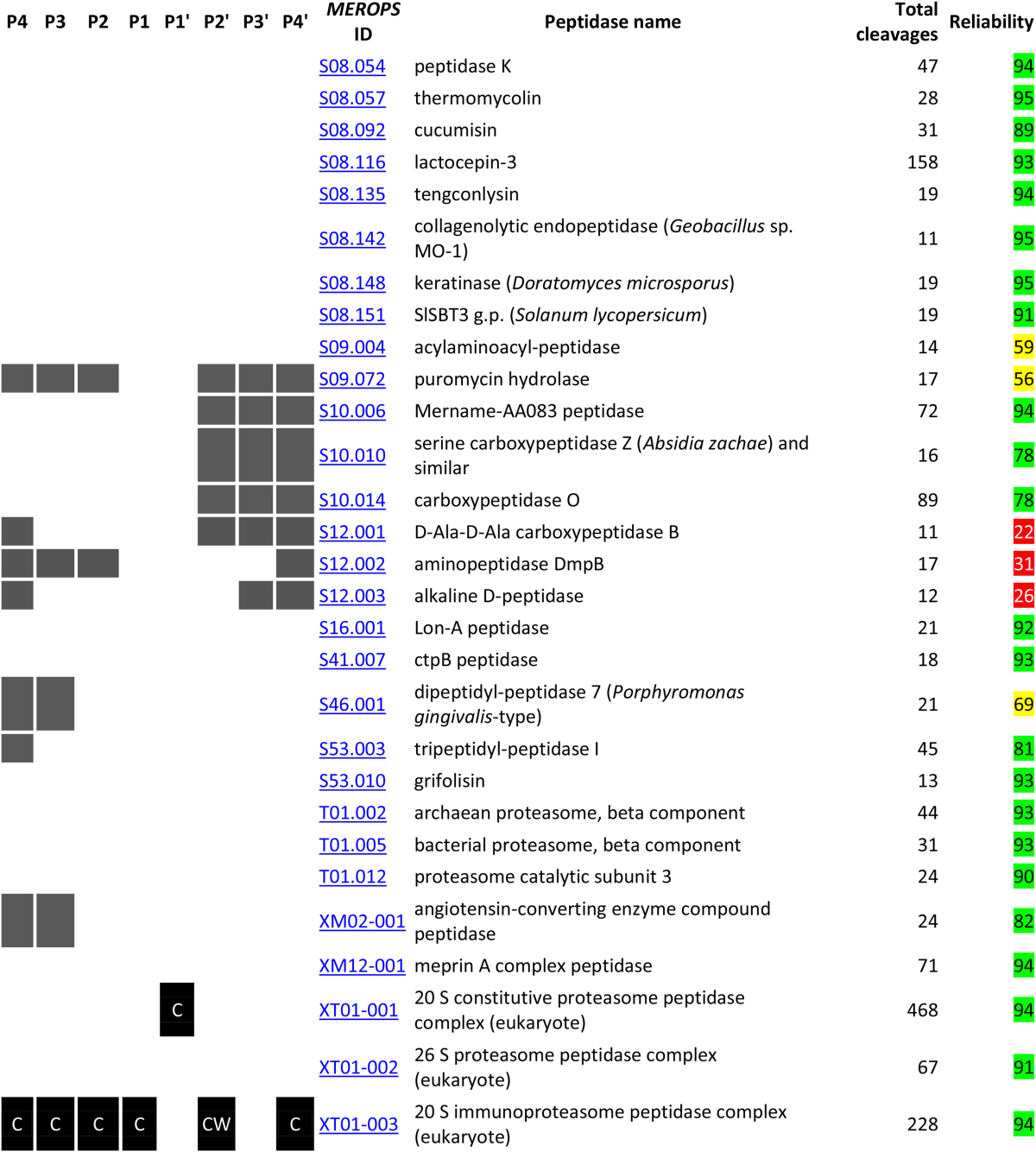
Peptidases with 10 or more known cleavages and peptidase specificity derived from substrate cleavages in the *MEROPS* collection. For each peptidase with ten or more known substrate cleavages are shown. Peptidases are arranged by specificity (number of binding pockets, then preference in each binding pocket in the order P4 to P4′, then by amino acid in alphabetical order) and then by MEROPS identifier. For each peptidase, the MEROPS identifier, the recommended peptidase name, the number of substrate cleavages, and preferences for binding pockets P4 to P4′ are shown. The brighter the shade of green, the greater the preference; five shades are shown ranging from darkest green (50–59% of substrates) to brightest green (90% or greater of substrates). Up to two amino acids are shown in a binding pocket where a preference occurs. Where the preference is for any of a group of amino acids, or the preference for the group is greater than that for a single or amino acid, the following symbols are shown: λ (aliphatic: Ile, Leu, Val), @ (aromatic: Phe, Trp, Tyr), + (acidic: Asp, Glu), − (basic: Arg, His, Lys), Σ (small: Ala, Cys, Gly, Ser) and Ω (other: Asn, Gln, Met, Pro, Thr). Where no preference for an amino acid or group of amino acids exists, and where there are 200 or more cleavages, up to two amino acids that are not acceptable in a binding pocket are shown as white text on a black background. For exopeptidases which act at N- and C-termini of proteins, no residue may be possible in some binding pockets and in these cases the binding pockets are shaded grey. Binding pockets shaded black or grey are ignored for the ordering of items in the table. The “Reliability score” is the percentage difference calculated by counting all the differences between substrates for the same enzyme, dividing the total differences by the number of comparisons times the number of residues P4–P4′ considered, and multiplying by 100. Reliability scores of 75% or greater difference are highlighted in green; scores 50% or greater in yellow, and scores of less than 50% in red. See text for details.

**Table 5:** An example of a file for submission to the Analyse Substrates service.

MEROPS ID	UniProt	Cleavage position
A01.004	P05067	671
A01.009	P05067	705
A01.009	P05067	713
A01.009	P05067	714
A01.009	P05067	719
A01.009	P05067	720
A01.041	P05067	690
A01.041	P05067	691
A22.001	P05067	711
A22.001	P05067	713
A22.001	P05067	714
C01.060	P05067	704
C01.060	P05067	708
C01.060	P05067	711
C01.084	P05067	685
C01.084	P05067	685
C01.084	P05067	685
C01.084	P05067	689
C01.084	P05067	690
C01.084	P05067	690
C14.003	P05067	739
C14.005	P05067	672
C14.005	P05067	739
M02.001	P05067	711
M10.003	P05067	687
M10.003	P05067	705
M10.003	P05067	706
M10.004	P05067	687
M10.004	P05067	691
M10.004	P05067	694
M10.004	P05067	701
M10.004	P05067	704
M10.004	P05067	705
M10.014	P05067	579
M10.014	P05067	687
M10.016	P05067	463
M10.016	P05067	579
M10.016	P05067	622
M10.016	P05067	685
M10.017	P05067	685
M10.017	P05067	687

**Table 6:** Results from the Analyse Substrates service.

MEROPS identifier	Total cleavages known	Substrate UniProt accession	Homologues	Cleaved at	P4 count	P3 count	P2 count	P1 count	P1′ count	P2′ count	P3′ count	P4′ count
A01.004	24	P05067	352	671	10	7	25	5	0	0	0	1
A01.009	897	P05067	352	705	0	0	0	0	0	0	1	0
A01.009	897	P05067	352	713	0	0	1	1	6	5	5	5
A01.009	897	P05067	352	714	0	1	1	6	5	5	5	5
A01.009	897	P05067	352	719	0	0	0	0	0	1	1	1
A01.009	897	P05067	352	720	0	0	0	0	1	1	1	3
A01.041	33	P05067	352	690	0	2	2	1	1	3	1	10
A01.041	33	P05067	352	691	2	2	1	1	1	1	1	1
A22.001	16	P05067	352	711	1	0	0	1	1	2	6	6
A22.001	16	P05067	352	713	4	1	1	2	6	5	5	5
A22.001	16	P05067	352	714	0	1	1	6	5	5	5	8
C01.060	632	P05067	352	704	0	0	0	0	0	0	0	1
C01.060	632	P05067	352	708	0	0	0	1	0	0	0	1
C01.060	632	P05067	352	711	1	0	0	0	1	1	5	5
C01.084	19	P05067	352	685	1	0	30	1	6	1	3	3
C01.084	19	P05067	352	685	1	0	30	1	6	1	3	3
C01.084	19	P05067	352	685	1	0	30	1	6	1	3	3
C01.084	19	P05067	352	689	0	1	3	2	2	6	4	2
C01.084	19	P05067	352	690	1	3	3	1	5	4	4	8
C01.084	19	P05067	352	690	1	3	3	1	5	4	4	8
C14.003	651	P05067	352	739	1	1	4	4	3	3	3	3
C14.005	201	P05067	352	672	7	5	4	16	0	0	0	0
C14.005	201	P05067	352	739	1	1	4	4	3	3	3	3
M02.001	5	P05067	352	711	1	1	4	1	1	2	173	173
M10.003	3417	P05067	352	687	2	0	0	0	0	1	1	1
M10.003	3417	P05067	352	705	0	0	0	0	0	0	1	0
M10.003	3417	P05067	352	706	0	0	0	0	0	1	0	0
M10.004	369	P05067	352	687	2	0	0	0	0	1	1	1
M10.004	369	P05067	352	691	0	1	1	1	1	1	1	1
M10.004	369	P05067	352	694	0	0	0	0	0	0	0	0
M10.004	369	P05067	352	701	0	0	0	0	0	0	0	0
M10.004	369	P05067	352	704	0	0	0	0	0	0	0	1
M10.004	369	P05067	352	705	0	0	0	0	0	0	1	0
M10.014	132	P05067	352	579	0	0	0	0	0	0	0	0
M10.014	132	P05067	352	687	11	0	0	0	0	1	1	1
M10.016	20	P05067	352	463	0	0	0	0	0	0	0	9
M10.016	20	P05067	352	579	7	0	18	0	2	4	0	1
M10.016	20	P05067	352	622	13	6	19	2	1	6	11	6
M10.016	20	P05067	352	685	0	0	11	1	5	0	0	2
M10.017	27	P05067	352	685	0	0	11	1	5	0	0	2
M10.017	27	P05067	352	687	11	1	0	0	0	3	1	1
